# Dichotomous mechanistic behavior in Narasaka–Heck cyclizations: electron rich Pd-catalysts generate iminyl radicals†
†Electronic supplementary information (ESI) available: Experimental procedures and characterisation data for all compounds are provided. CCDC 1429194. For ESI and crystallographic data in CIF or other electronic format see DOI: 10.1039/c5sc04037j


**DOI:** 10.1039/c5sc04037j

**Published:** 2015-12-01

**Authors:** Nicholas J. Race, Adele Faulkner, Megan H. Shaw, John F. Bower

**Affiliations:** a School of Chemistry , University of Bristol , Bristol , BS8 1TS , UK . Email: john.bower@bris.ac.uk ; Fax: +44 (0)117 925 1295

## Abstract

Pd-catalyzed cyclizations of oxime esters with pendant alkenes are subject to an unusual ligand controlled mechanistic divergence. Pd-systems modified with electron deficient phosphines (*e.g.* P(3,5-(CF_3_)_2_C_6_H_3_)_3_) promote efficient aza-Heck cyclization, wherein C–N bond formation occurs *via* alkene imino-palladation. Conversely, electron rich ligands, such as P(*t*-Bu)_3_, cause deviation to a SET pathway and, in these cases, C–N bond formation occurs *via* cyclization of an iminyl radical. A series of mechanistic experiments differentiate the two pathways and the scope of the hybrid organometallic radical cyclization is outlined. This study represents a rare example in Pd-catalysis where selection between dichotomous mechanistic manifolds is facilitated solely by choice of phosphine ligand.

## Abstract 2

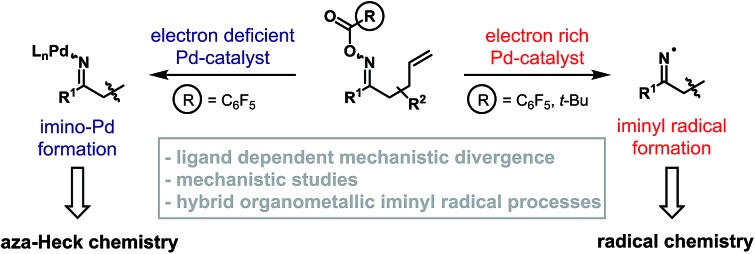
Pd-catalyzed cyclizations of oxime esters with pendant alkenes undergo ligand controlled mechanistic divergence. Electron deficient phosphines promote aza-Heck cyclization; electron rich systems favour a SET pathway. Mechanistic experiments differentiate the two manifolds.

## Introduction

**Scheme 1:**
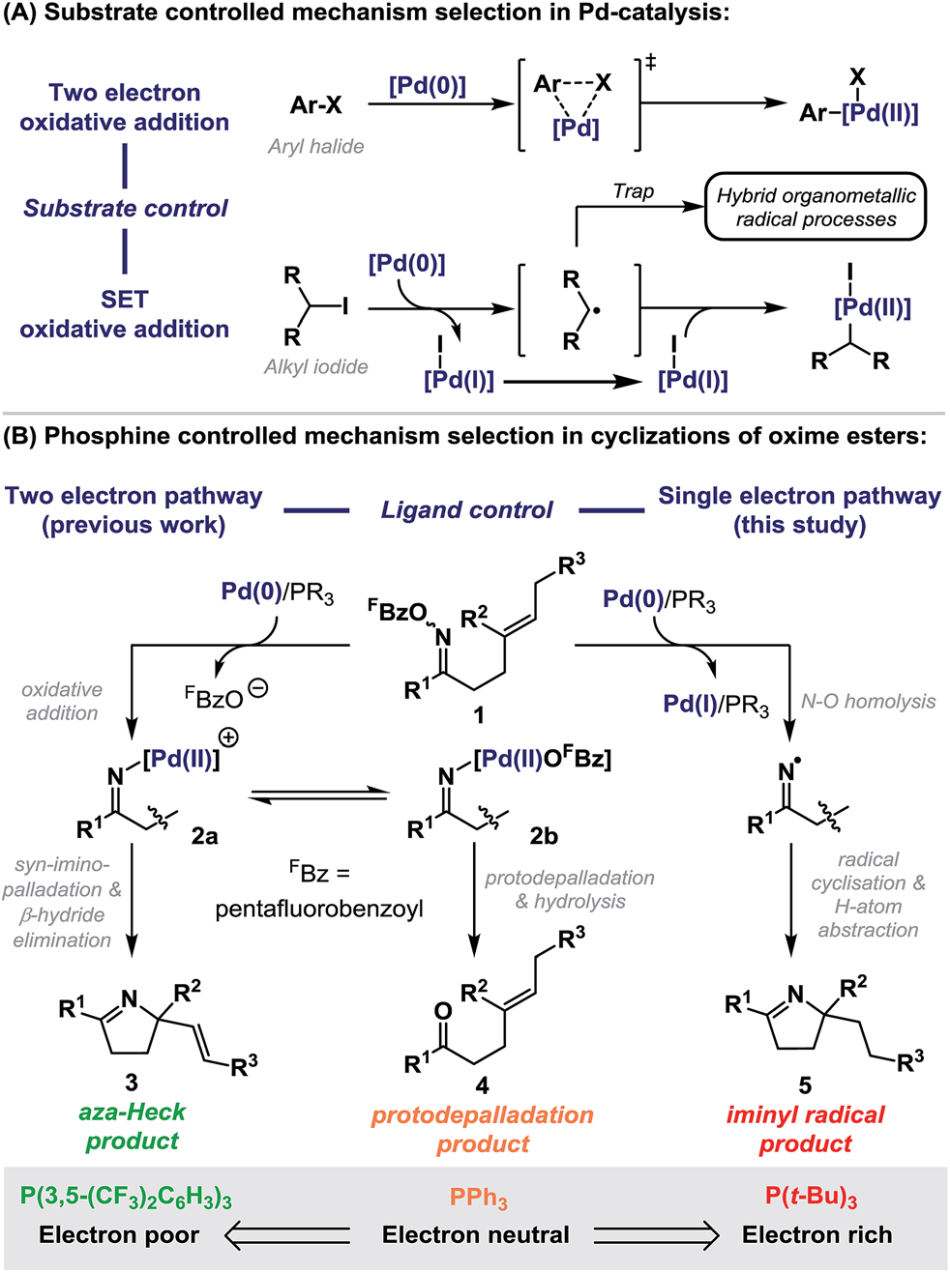


Palladium-catalyzed processes are fundamental to organic synthesis, and it is estimated that one in five C–C bond formations employed in commercial syntheses of new drugs are reliant on this technology.^1^ Significant and continuing efforts are focused on the development of diverse phosphine ligands to enhance key mechanistic steps, such as oxidative addition.^2^ Consequently, the overall efficiency of a given process is strongly dependent on the exact choice of P-based ligand. However, cases where this choice causes deviation from common two electron redox processes to one electron, radical-based pathways are rare.^3^ This is despite the well documented, but underappreciated propensity of Pd(0)-systems to undergo single electron transfer (SET) oxidative addition in certain contexts,^4^ and the emergence of a series of hybrid organometallic-radical methodologies that invoke the intermediacy of Pd(i)-complexes (Scheme 1A).^5^

We have reported a range of aza-Heck methodologies that involve oxidative addition of Pd(0)-catalysts into the N–O bond of *O*-pentafluorobenzoyl oxime esters **1** (Scheme 1B, two electron pathway).^6^–^8^ Addition of the resulting imino-Pd intermediate **2a** across a pendant alkene leads to aza-Heck (**3**)^6^,^7^ or cascade products.^8^ For these processes, electron deficient ligand systems, especially P(3,5-(CF_3_)_2_C_6_H_3_)_3_, are most effective, as they enhance migratory insertion and suppress protodepalladation of **2a**/**b**; this latter pathway leads to the corresponding ketone **4** and predominates with electron neutral ligand systems, such as PPh_3_.^7^ In this report we disclose that electron rich phosphines (*e.g.* P(*t*-Bu)_3_) do not lead to Heck type products, but instead promote exclusive access to radical manifolds (Scheme 1B, single electron pathway). This has important implications for processes reliant upon the oxidative addition of Pd(0)-catalysts into oxime ester N–O bonds. Indeed, in addition to aza-Heck reactions,^6^,^7^ this catalysis platform has enabled diverse methodologies, including alkene 1,2-carboaminations,^8^ aryl C–H aminations,^9^ alkene aziridinations,^10^ alkene 1,2-iodoaminations,^11^ benzyne 1,2-aminofunctionalizations,^12^ and C–C bond activations.^13^ Furthermore, this study provides convenient and unique access to iminyl radical chemistry,^14^ and in broader terms, represents a rare example in Pd-catalysis where selection between dichotomous mechanistic manifolds is facilitated solely by choice of phosphine ligand.^3^

## Results and discussion

Under optimized aza-Heck conditions, which use P(3,5-(CF_3_)_2_C_6_H_3_)_3_ as ligand, cyclization of *O*-pentafluorobenzoyl oxime ester **1a** to alkene **3a** occurs in 93% yield (Table 1, entry 3).7*a* The *O*-pentafluorobenzoyl group is important, as, following oxidative addition, the pentafluorobenzoate leaving group undergoes facile protodecarboxylation to C_6_F_5_H, which drives access to cationic intermediate **2a**, as required for cyclization.^8^ When PPh_3_ was used as ligand, a 30% yield of **3a** was achieved (entry 1) and the mass balance consisted predominantly of the corresponding ketone, which likely arises *via* protodepalladation of intermediate **2b**. For *O*-pivaloyl oxime ester **1b**, cyclization to **3a** was not observed using either P(3,5-(CF_3_)_2_C_6_H_3_)_3_ or PPh_3_, and, in both cases, the only identifiable product was the corresponding ketone. Here, the issue is likely that the pivalate leaving group does not dissociate readily after oxidative addition to provide access to key cationic intermediate **2a**. Thus, effective aza-Heck cyclization requires both an *O*-pentafluorobenzoyl oxime ester and an electron deficient phosphine ligand, whereas protodepalladation predominates using electron neutral phosphines and/or weakly dissociating leaving groups. Exposure of either **1a** or **1b** to the electron rich Pd-system (d*t*-bpf)PdCl_2_ did not lead to aza-Heck product **3a** or significant quantities of ketone. Instead, adduct **5a** was isolated in 72% yield in both cases (entries 4 and 5). **5a** is a formal ‘reductive’ aza-Heck product, however, as outlined later, this likely arises *via* a Pd(0)-triggered radical-based cyclization. The situation appears to be general for a range of electron rich phosphines, including P(*t*-Bu)_3_, and PCy_3_, and other classes of strong donor ligand, such as N-heterocyclic carbenes (entries 6–10). The use of common hydride sources, such as HCO_2_H, in conjunction with d*t*-bpf as ligand was detrimental to the yield of **5a**. However, common hydrogen atom donors, such as 1,4-cyclohexadiene (1,4-CHD) and γ-terpinene enhanced cyclization efficiency, with the latter providing **5a** in 88% yield (entries 12 and 13). These observations provided early evidence for a radical based pathway.^15^

**Table 1:** Ligand effects on the cyclization of oxime esters **1a**/**b**

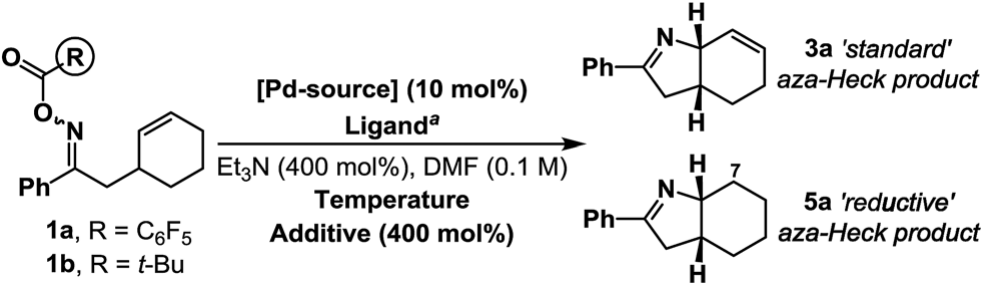						
Entry	R	Pd-source/ligand	Temp/°C	Additive	**3a** ^*d*^ (%)	**5a** ^*d*^ (%)
1	C_6_F_5_	Pd_2_(dba)_3_/PPh_3_	100	None	30	<5
2	C_6_F_5_	Pd_2_(dba)_3_/P(3,5-(CF_3_)_2_C_6_H_3_)_3_	120	None	90	<5
3	C_6_F_5_	Pd_2_(dba)_3_/P(3,5-(CF_3_)_2_C_6_H_3_)_3_^*c*^	60	None	93	<5
4	C_6_F_5_	(d*t*-bpf)PdCl_2_^*b*^	120	None	<5	72
5	*t*-Bu	(d*t*-bpf)PdCl_2_^*b*^	120	None	<5	72
6	*t*-Bu	Pd_2_(dba)_3_/S-Phos	120	None	<5	10
7	*t*-Bu	Pd_2_(dba)_3_/P(1-Ad)_2_*n*-Bu	120	None	<5	29
8	*t*-Bu	Pd_2_(dba)_3_/P(Cy)_3_	120	None	<5	30
9	*t*-Bu	Pd_2_(dba)_3_/P(*t*-Bu)_3_	120	None	<5	50
10	*t*-Bu	PEPPSI-IPr	120	None	<5	27
11	*t*-Bu	(d*t*-bpf)PdCl_2_^*b*^ ^,^^*c*^	70	None	<5	67
12	*t*-Bu	(d*t*-bpf)PdCl_2_^*b*^ ^,^^*c*^	70	1,4-CHD	<5	80
13	*t*-Bu	(d*t*-bpf)PdCl_2_^*b*^ ^,^^*c*^	70	γ-Terpinene	<5	88

^*a*^1 : 2 [Pd] : ligand for monodentate systems, 1 : 1 [Pd] : ligand for bidentate systems.

^*b*^d*t*-bpf = 1,1′-bis(di-*tert*-butylphosphino)ferrocene.

^*c*^5 mol% Pd/L used.

^*d*^Isolated yield.

**Scheme 2:**
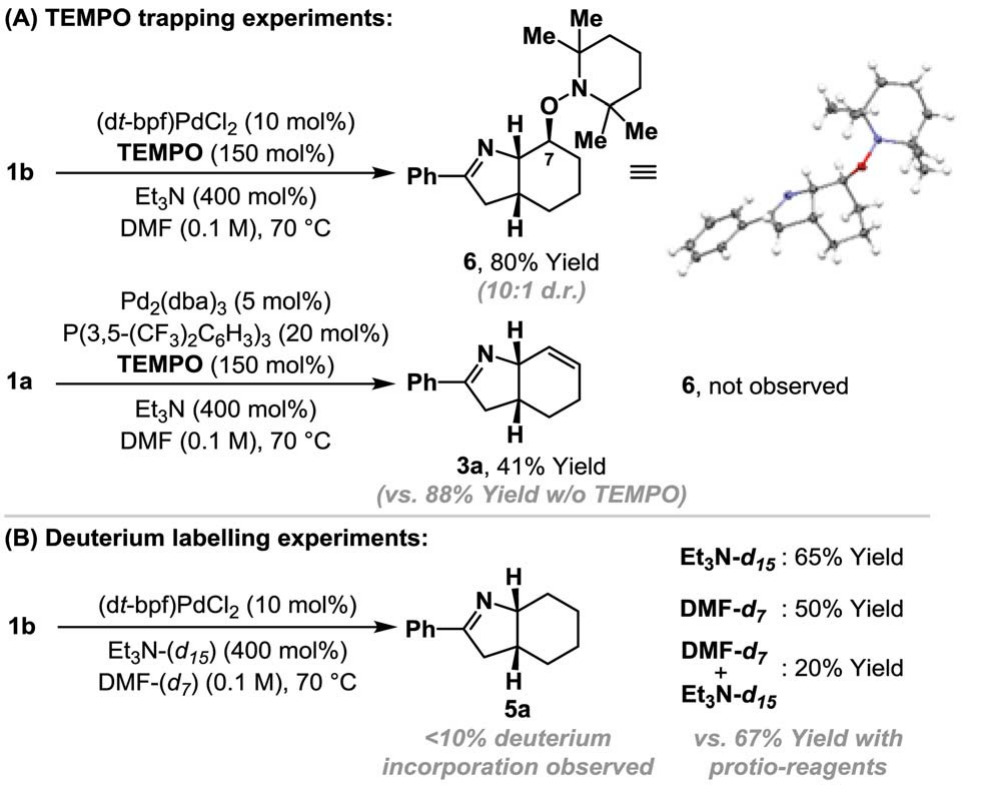
Preliminary mechanistic studies.

Cyclization of **1b** under optimized ‘reductive’ aza-Heck conditions, but in the presence of TEMPO (150 mol%) provided trapping adduct **6** in 80% yield and 10 : 1 d.r.; the structure of the major diastereomer was confirmed by single crystal X-ray diffraction (Scheme 2A).^16^ An analogous trapping experiment on **1a**, using optimized ‘standard’ aza-Heck conditions, did not generate **6**, and ‘standard’ aza-Heck product **3a** was formed in 41% yield. The formation of **6** is consistent with cyclization to generate an alkyl radical at C7 during the conversion of **1a**/**b** to **5a**, however, in the absence of exogenous hydrogen atom donors, the source of reductant is unclear (*cf.*Table 1, entry 11 *vs.* 13). Cyclization of **1b**, under the conditions outlined in Table 1, entry 11, using DMF-d_7_/Et_3_N or DMF-d_7_/Et_3_N-d_15_ did not result in appreciable levels of deuterium incorporation in adduct **5a** (Scheme 2B). However, in both cases, the yield of **5a** was lower than when solely *protio*-reagents were used. Overall, these observations implicate the feasibility of hydrogen atom abstraction from either DMF, Et_3_N, or other components of the reaction system (*e.g.***1a**/**b** or **5a**).^17^

**Scheme 3:**
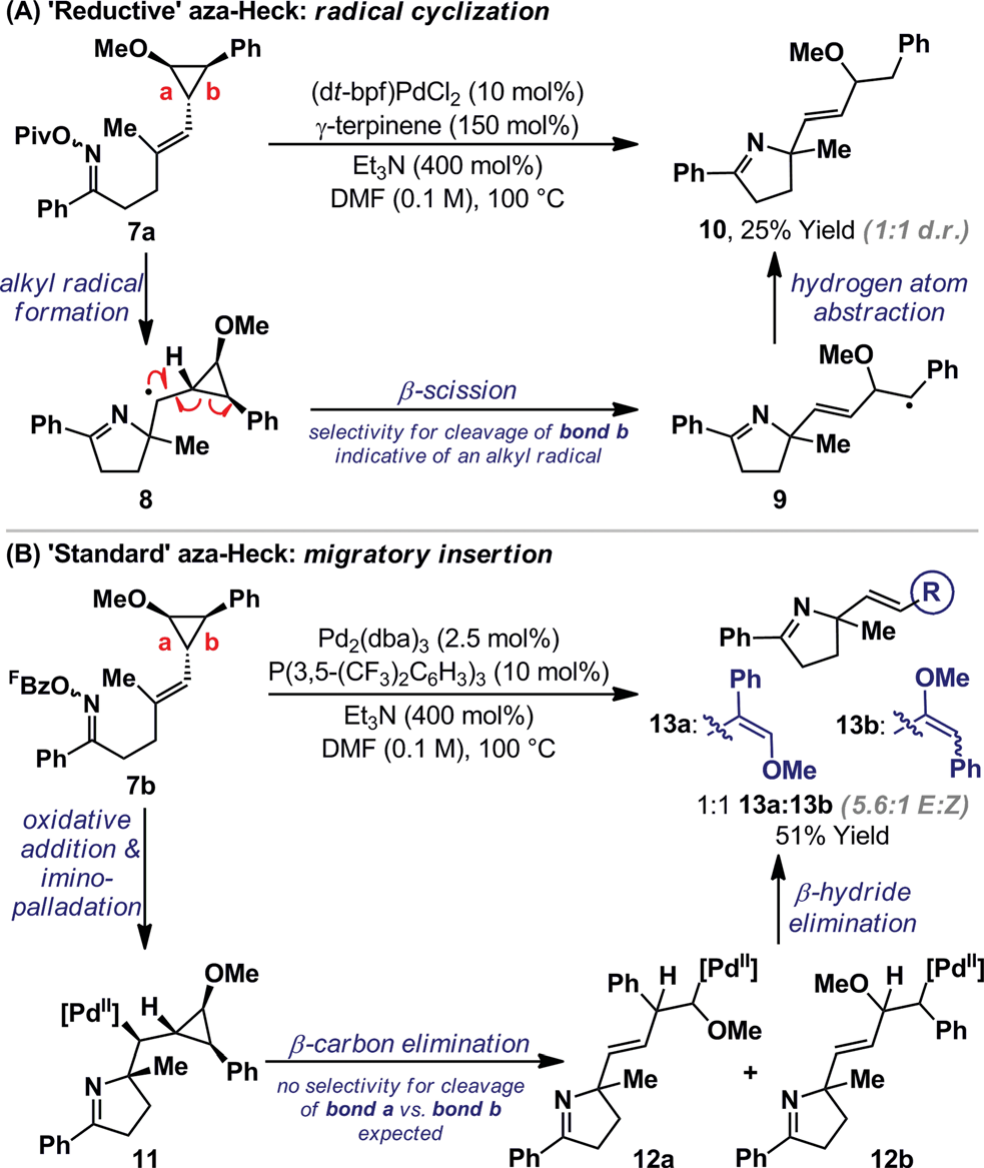
Cyclopropane mechanistic experiments.

TEMPO can trigger radical based pathways when employed as a probe in Pd-catalyzed processes.^18^ Consequently, the studies outlined in Scheme 2A are not definitive proof for the intermediacy of an alkyl radical during the cyclization of **1a**/**b** to **5a**. To provide further evidence, experiments based on Newcomb's radical probe were devised.^19^ Exposure of *O*-pivaloyl oxime ester **7a** to ‘reductive’ aza-Heck conditions resulted in the formation of dihydropyrrole **10** in 25% yield and as the only observable product (Scheme 3A); the regioselectivity of cyclopropane cleavage was determined by 2D NMR analysis (see the ESI†). The sole formation of **10** is consistent with initial cyclization to alkyl radical **8**, which undergoes *selective* β-scission (*via* bond b) to generate stabilized benzylic radical **9**. Hydrogen atom abstraction from γ-terpinene then yields **10**. Cyclization of *O*-pentafluorobenzoyl oxime ester **7b**, under optimized ‘standard’ aza-Heck conditions, resulted in a 51% yield and 1 : 1 ratio of unstable dihydropyrroles **13a**/**b** (Scheme 3B); the latter was formed as a 5.6 : 1 mixture of alkene isomers. This result is consistent with an imino-palladation pathway, wherein cyclization generates alkyl-Pd(ii) intermediate **11**. This is not expected to have significant radical (or carbocationic) character, such that *non-selective* β-carbon elimination (to **12a**/**b**) ensues en route to **13a**/**b**.^20^ Products of β-hydride elimination from alkyl-Pd(ii) intermediate **11** were not observed. The studies in Scheme 3 provide strong evidence for a radical-based pathway to **5a** and an imino-palladation pathway to **3a**.

**Scheme 4:**
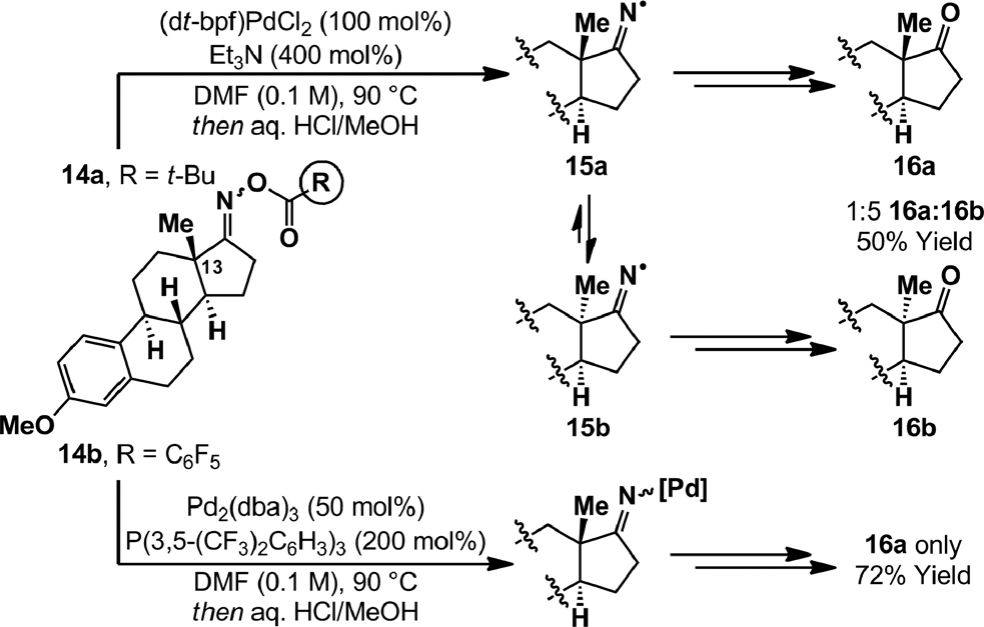
Estrone mechanistic experiments.

The most likely pathway to alkyl radical **8** is *via* cyclization of an iminyl radical. The generation of these from oxime esters is documented widely,^14^ however palladium-catalyzed conditions have not been reported. The oxidative addition of PCy_3_ ligated Pd(0)-systems into oxime ester N–O bonds is known, and both Hartwig and Stahl have characterized associated imino-Pd(ii) complexes by X-ray diffraction.^9^,^21^ However, little is known about the exact nature of the process and further insights were warranted given that, in the present study, PCy_3_ leads to radical cyclization product **5a** (Table 1, entry 8). To examine this aspect we have employed estrone derived oxime esters **14a**/**b** (Scheme 4); Zard and co-workers have shown that iminyl radicals derived from substrates of this type lead to inversion of the C13 stereocenter.^22^ Exposure of a DMF solution of *O*-pivaloyl oxime ester **14a** to (d*t*-bpf)PdCl_2_ (100 mol%) and Et_3_N (400 mol%), in the absence of γ-terpinene, resulted in complete consumption of starting material after 15 minutes at 90 °C. After hydrolytic work-up, a 1 : 5 mixture of estrone derivatives **16a** and **16b** was isolated in 50% yield. The formation of **16b** is consistent with SET from Pd(0) to generate iminyl radical **15a**, which undergoes reversible β-scission (*via* the corresponding nitrile) to afford thermodynamically favored diastereomer **15b**. Incomplete inversion of the methyl-substituted stereocenter may be due to quenching of iminyl radical **15a** by either hydrogen atom abstraction (from elsewhere in the system) or recombination with Pd(i). An analogous experiment on *O*-pentafluorobenzoyl oxime ester **14b**, using stoichiometric Pd(0)/P(3,5-(CF_3_)_2_C_6_H_3_)_3_, generated ketone **16a** exclusively in 72% yield. Overall, these results suggest that N–O oxidative addition involving electron rich Pd(0)-systems has significant SET character, whereas electron poor systems insert *via* a two electron redox pathway. We note that, in certain cases, oxidative addition of Pd(0)-catalysts into alkyl-iodide bonds has been shown to proceed *via* a SET pathway;^4^ these observations established that the nature of the substrate can change the mechanistic pathway from that commonly observed for aryl halides (see Scheme 1A). However, the results described in the present study are unique examples where analogous mechanistic deviations are achieved simply by altering the ancillary ligand on Pd.^3^,^23^

**Scheme 5:**
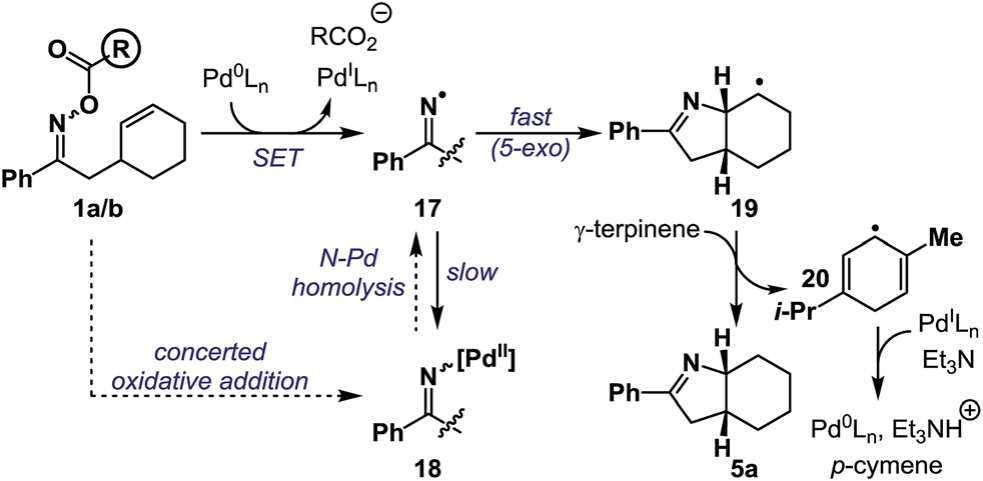
A working mechanistic hypothesis.

Based on the above results, a plausible mechanism for the conversion of **1a**/**b** to **5a** is outlined in Scheme 5. Single electron transfer from Pd(0) to oxime ester **1a**/**b** results in N–O cleavage to generate Pd(i) and iminyl radical **17**. Studies by the groups of Hartwig and Stahl^9^,^21^ suggest that, in principle, recombination could generate imino-Pd(ii) intermediate **18**, however, this work was conducted on systems without an alkene acceptor. Consequently, in the present case, recombination is ‘interrupted’ by competing and fast 5-*exo* cyclization to generate alkyl radical **19**, which is quenched by hydrogen atom abstraction from γ-terpinene to afford product **5a** and bis-allylic radical **20**. Hydrogen atom transfer from **20** to Pd(i) generates *p*-cymene and a Pd(ii)-hydride, which undergoes base-induced reductive elimination to Pd(0) to close the catalytic cycle. At the present stage, iminyl radical generation *via* formation and subsequent N–Pd homolysis of imino-Pd(ii) intermediate **18** cannot be discounted. The proposed hybrid organometallic radical mechanism is unusual and adds to a growing body of processes that use late transition metal catalysts to accomplish classical radical processes.^5^

The ability to promote iminyl radical cyclizations using a Pd(0)-catalyst, in combination with γ-terpinene, represents a novel and potentially useful approach to alkene hydroamination. Related iminyl-radical based protocols^14^ often require specialized (and costly) *O*-activating groups (*e.g. O*-Ph)14c,f or toxic (*e.g.* Bu_3_SnH/AIBN)14*d* and/or operationally challenging conditions (*e.g.* UV/visible light irradiation) that are difficult to scale-up.14e,f In light of the efficiency of the conversion of **1b** to **5a**, we have conducted a preliminary examination of the scope using a range of pivaloyl oxime esters **21a–f** (Table 2). Aryl- and alkyl-substituted oximes esters are tolerated and cyclization occurred in moderate to excellent yields using a range of alkene acceptors. For **21b**, cyclization of a 1 : 1 mixture of diastereomers at C2 provided product **22b** in high diastereopurity, likely as a result of post-cyclization epimerization to the thermodynamically favored diastereomer.7*a* The results outlined in Table 2 show that the present protocol provides a useful entry to iminyl radical chemistry.^14^

**Table 2:** Iminyl radical cyclization scope^*a*^

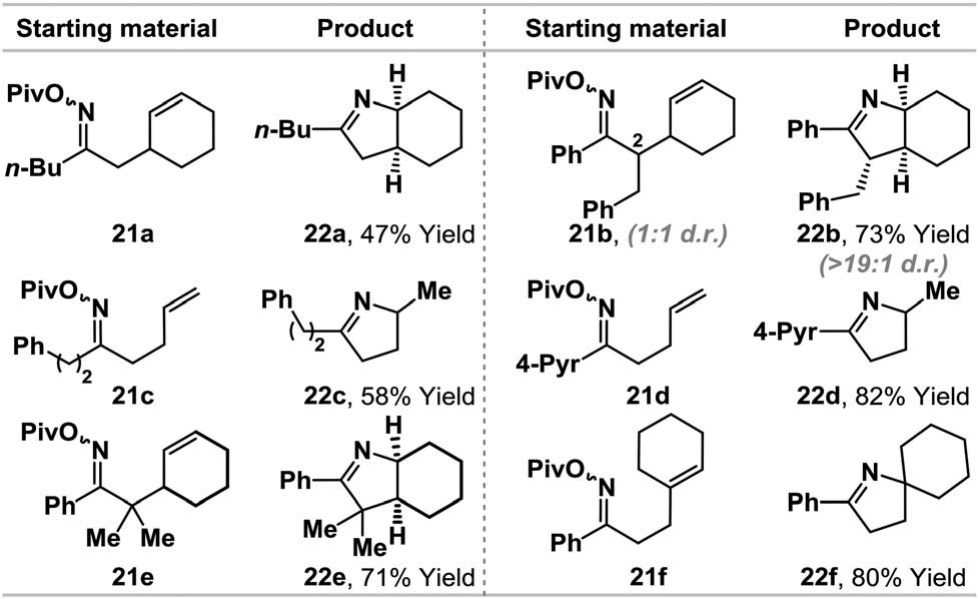

^*a*^Cyclizations were run under the following conditions: (d*t*-bpf)PdCl_2_ (5 mol%), γ-terpinene (400 mol%), Et_3_N (400 mol%), DMF (0.1 M), 70–90 °C, 16–24 h. Full details are given in the ESI.

## Conclusions

In summary, we demonstrate that Pd-catalyzed cyclizations of oxime esters can be partitioned between dichotomous mechanistic manifolds solely through choice of phosphine ligand. Electron rich phosphines promote SET-type oxidative addition, which is ‘interrupted’ at the stage of an iminyl radical to provide hybrid organometallic radical C–N bond forming cyclizations. For electron poor phosphines, N–O oxidative addition proceeds *via* a ‘conventional’ two electron pathway to generate directly imino-palladium intermediates, which engage pendant alkenes in a Heck-like manner. These mechanistic insights will guide ongoing efforts in our laboratory aimed at providing a general aza-Heck protocol. A wide range of processes are dependent upon aza-Pd(ii) intermediates generated by N–O oxidative addition,^6^–^13^ and, as such, the studies outlined here are likely to be of importance beyond the immediate area of aza-Heck cyclizations.

## Note added after first publication

This article replaces the version published on 1st December 2015, which contained errors in Scheme 2.

## Supplementary Material

Supplementary information

Crystal structure data
