# Supplementary material for: Dichotomous mechanistic behavior in Narasaka–Heck cyclizations: electron rich Pd-catalysts generate iminyl radicals
Source: Chem Sci. 2015 Dec 1;7(2):1508–13. doi: 10.1039/c5sc04037j (PMC5963672; doi:10.1039/c5sc04037j)
Supplement: Supplementary file 1 [file SC-007-C5SC04037J-s001.pdf]

Electronic Supplementary Material (ESI) for Chemical Science.
This journal is © The Royal Society of Chemistry 2015

# Dichotomous Mechanistic Behavior in Narasaka-Heck Cyclizations: Electron Rich Pd-Catalysts Generate Iminyl Radicals

Nicholas J. Race, Adele Faulkner, Megan H. Shaw, and John F. Bower*

*School of Chemistry, University of Bristol, Bristol, BS8 1TS, UK*

## Electronic Supplementary Information

### Table of Contents

General Experimental Details ---------------------------------------------------------------------- 2

Experimental Procedures and Data ---------------------------------------------------------------- 3

Copies of $^{1}$H and $^{13}$C NMR spectra for novel compounds ------------------------------------- 15

References ------------------------------------------------------------------------------------------ 31

**General Experimental Details.** Starting materials sourced from commercial suppliers were used as received unless otherwise stated. Dry solvents, where necessary, were obtained by distillation using standard procedures or by passage through a column of anhydrous alumina using equipment from Anhydrous Engineering based on the Grubb's design. The removal of solvents *in vacuo* was achieved using both a Büchi rotary evaporator (bath temperatures up to 40 °C) at a pressure of either 15 mmHg (diaphragm pump) or 0.1 mmHg (oil pump), as appropriate, and a high vacuum line at r.t.. Reactions requiring anhydrous conditions were run under an atmosphere of dry nitrogen or argon; glassware, syringes and needles were either flame dried immediately prior to use or placed in an oven (200 °C) for at least 2 h and allowed to cool either in a desiccator or under an atmosphere of nitrogen or argon; liquid reagents, solutions or solvents were added *via* syringe through rubber septa; solid reagents were added *via* Schlenk type adapters. Commercially available Merck Kieselgel $60F_{254}$ aluminium backed plates were used for TLC analysis. Visualization was achieved by either UV fluorescence or basic $KMnO_4$ solution and heat. Flash column chromatography (FCC) was performed using silica gel (Aldrich 40-63 µm, 230-400 mesh). The crude material was applied to the column as a solution in $CH_2Cl_2$ or by pre-adsorption onto silica, as appropriate. Melting points were determined using a Reichert melting point table and temperature controller and are uncorrected. Infra-red spectra were recorded in the range 4000-600 $cm^{-1}$ on a Perkin Elmer Spectrum either as neat films or solids compressed onto a diamond window. NMR spectra were recorded using either a Varian 400 MHz or JOEL ECS 400 MHz spectrometer. Chemical shifts (δ) are quoted in parts per million (ppm), coupling constants (*J*) are given in Hz to the nearest 0.5 Hz. Other abbreviations used are s (singlet), d (doublet), t (triplet), m (multiplet) and br. (broad). $^{1}H$ and $^{13}C$ NMR spectra were referenced to the appropriate residual solvent peak. Assignments of $^{1}H$ NMR and $^{13}C$ NMR signals were made, where possible, using COSY, DEPT, HMQC, HMBC and NOE experiments. Where mixtures of isomers (*e.g.* diastereomers and/or rotamers) have been characterized together, they are referred to as A and B. *Numbering systems for NMR signal assignments are specified on the structure and are not related to those used for the compound names.* Mass spectra were determined by the University of Bristol mass spectrometry service by either electron impact ($EI^+$) or chemical ionization ($CI^+$) using a Fisons VG Analytical Autospec spectrometer, or by electrospray ionization ($ESI^+$) using a Brüker Daltonics Apex IV spectrometer.

# Experimental procedures and Data.

## Ligand effects on the cyclization of oxime esters 1a/b

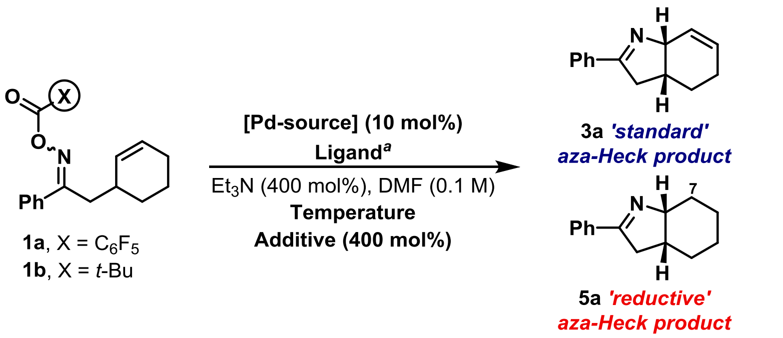


| Entry | X | Pd-source/Ligand | Temp/°C | Additive | 3a (%)[d] | 5a (%)[d] |
|---|---|---|---|---|---|---|
| 1 | $C_6F_5$ | $Pd_2(dba)_3/PPh_3$ | 100 | none | 30 | <5 |
| 2 | $C_6F_5$ | $Pd_2(dba)_3$/P(2-furyl)$_3$ | 100 | none | 38 | <5 |
| 3 | $C_6F_5$ | $Pd_2(dba)_3/P(4\text{-}FC_6H_4)_3$ | 100 | none | 76 | <5 |
| 4 | $C_6F_5$ | $Pd_2(dba)_3/P(4\text{-}CF_3C_6H_4)_3$ | 100 | none | 85 | <5 |
| 5 | $C_6F_5$ | $Pd_2(dba)_3/P(3,5\text{-}(CF_3)_2C_6H_3)_3$ | 120 | none | 90 | <5 |
| 6 | $C_6F_5$ | $Pd_2(dba)_3/P(3,5\text{-}(CF_3)_2C_6H_3)_3$[c] | 60 | none | 93 | <5 |
| 7 | $C_6F_5$ | $Pd_2(dba)_3/P(C_6F_5)_3$ | 100 | none | 10 | <5 |
| 8 | $C_6F_5$ | (d*t*-bpf)$PdCl_2$[b] | 120 | none | <5 | 72 |
| 9 | *t*-Bu | (d*t*-bpf)$PdCl_2$[b] | 120 | none | <5 | 72 |
| 10 | *t*-Bu | $Pd_2(dba)_3$/S-Phos | 120 | none | <5 | 10 |
| 11 | *t*-Bu | $Pd_2(dba)_3$/P(1-Ad)$_2$*n*-Bu | 120 | none | <5 | 29 |
| 12 | *t*-Bu | $Pd_2(dba)_3$/P(Cy)$_3$ | 120 | none | <5 | 30 |
| 13 | *t*-Bu | $Pd_2(dba)_3$/P(*t*-Bu)$_3$ | 120 | none | <5 | 50 |
| 14 | *t*-Bu | PEPPSI-*i*-Pr | 120 | none | <5 | 27 |
| 15 | *t*-Bu | (d*t*-bpf)$PdCl_2$[b,c] | 70 | none | <5 | 67 |
| 16 | *t*-Bu | (d*t*-bpf)$PdCl_2$[b,c] | 70 | $HCO_2H$ | <5 | <5 |
| 17 | *t*-Bu | (d*t*-bpf)$PdCl_2$[b,c] | 70 | *i*-PrOH | <5 | 61 |
| 18 | *t*-Bu | (d*t*-bpf)$PdCl_2$[b,c] | 70 | 1,4-CHD | <5 | 80 |
| 19 | *t*-Bu | (d*t*-bpf)$PdCl_2$[b,c] | 70 | γ-terpinene | <5 | 88 |

[a] 1:2 [Pd]:ligand for monodentate systems, 1:1 [Pd]:ligand for bidentate systems. [b] d*t*-bpf = 1,1'-bis(di-*tert*-butylphosphino)ferrocene. [c] 5 mol% Pd/L used. [d] Isolated yield.

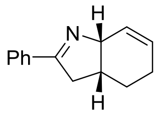


**(3a*R**,7a*S**)-2-Phenyl-3a,4,5,7a-tetrahydro-3*H*-indole 3a:** The optimized procedure for the cyclization of oxime ester **1a** to dihydropyrrole **3a** has been reported previously.[1]

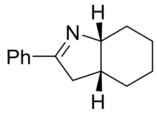


**(3a*R**,7a*R**)-2-Phenyl-3a,4,5,6,7,7a-hexahydro-3*H*-indole 5a:** An oven-dried reaction tube, fitted with magnetic stirrer, was charged with (d*t*-bpf)$PdCl_2$ (6.3 mg, 5 mol%) and oxime ester **1b** (58 mg,

0.19 mmol, 100 mol%). The tube was fitted with a rubber septum and purged with argon. DMF (1.90 mL, 0.1 M), $Et_3N$ (108 μL, 400 mol%) and γ-terpinene (124 μL, 400 mol%) were then added sequentially *via* syringe. The mixture was placed in a preheated heating block (70 °C) and stirred for 24 hours. The mixture was then cooled to room temperature and concentrated *in vacuo* (40 °C, approximately 1.0 mmHg). The residue was purified by FCC (4:1 hexane-EtOAc) to afford dihydropyrrole **5a** (34 mg, 88%) as a pale yellow oil. *The reaction proceeds with equal facility from oxime ester **1a**.* $\upsilon_{max}$ / $cm^{-1}$ (film) 2928, 2853, 1604, 1574, 1448; $\delta_H$ (400 MHz, $CDCl_3$) 7.90-7.83 (2H, m), 7.46-7.39 (3H, m), 4.02-3.96 (1H, m), 2.90 (1H, dddd, *J* = 16.0, 9.0, 7.0 and 2.0 Hz), 2.74 (1H, dddd, *J* = 16.0, 11.5, 4.5, and 1.0 Hz), 2.46-2.36 (1H, m), 1.96-1.89 (2H, m), 1.67-1.59 (1H, m), 1.54-1.44 (3H, m), 1.37-1.26 (2H, m); $\delta_C$ (100 MHz, $CDCl_3$) 173.5, 135.4, 130.3, 128.5, 127.5, 70.1, 41.6, 37.0, 29.3, 27.5, 23.1, 22.2; HRMS ($CI^+$) Found $[M+H]^+$: 200.1434, $C_{14}H_{18}N$ requires 200.1434.

*The relative stereochemistry of **5a** was assigned by analogy to compounds **3a**, **6** and **22b** (vide infra).*

## TEMPO trapping experiments

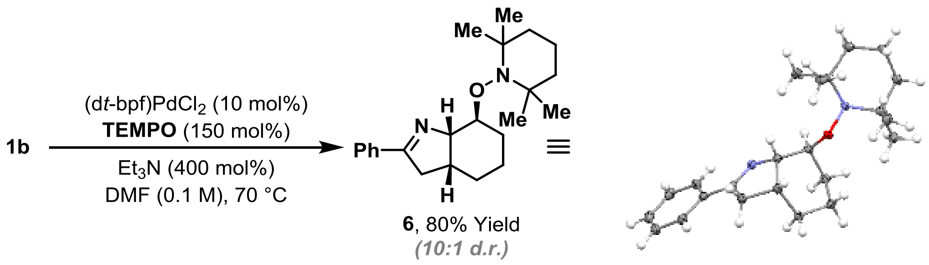


**(3a*R**,7*S**,7a*S**)-2-Phenyl-7-((2,2,6,6-tetramethylpiperidin-1-yl)oxy)-3a,4,5,6,7,7a-hexahydro-3*H*-indole 6:** An oven-dried reaction tube, fitted with magnetic stirrer, was charged with (d*t*-bpf)$PdCl_2$ (12.8 mg, 10 mol%), oxime ester **1b** (58 mg, 0.19 mmol, 100 mol%) and TEMPO (45 mg, 0.29 mmol, 150 mol%). The tube was fitted with a rubber septum and purged with argon. DMF (1.90 mL, 0.1 M) and $Et_3N$ (108 μL, 400 mol%) were then added sequentially *via* syringe. The mixture was placed in a preheated heating block (70 °C) and stirred for 24 hours. The mixture was then cooled to room temperature and concentrated *in vacuo* (40 °C, approximately 1.0 mmHg). The residue was purified by FCC (8:1 hexane-EtOAc) to afford dihydropyrrole **6** (54 mg, 80%, 10:1 d.r.) as a colorless, crystalline solid. Under these conditions, compound **5a** was not detected. $\upsilon_{max}$ / $cm^{-1}$ (film) 2935, 1604, 1571, 1447; $\delta_H$ (400 MHz, $CDCl_3$) 7.90-7.85 (2H, m), 7.45-7.35 (3H, m), 4.34-4.25 (1H, m), 4.21-4.15 (1H, m), 2.95 (1H, ddd, *J* = 16.0, 7.0, 2.0), 2.69 (1H, ddd, *J* = 16.0, 4.0, 1.0), 2.65-2.53 (1H, m), 1.84-1.73 (1H, m), 1.73-1.26 (11H, m), 1.23 (6H, s), 1.19 (6H, s); $\delta_C$ (100 MHz, $CDCl_3$) 173.5, 135.2, 130.4, 128.5, 127.7, 80.9, 73.7, 60.1, 42.1, 40.6, 36.1, 34.6, 27.8, 27.3 (2 signals), 20.6, 18.7, 17.4; HRMS ($ESI^+$) Found $[M+H]^+$: 355.2760, $C_{23}H_{35}N_2O$ requires 355.2744.

*The structure of the major diastereomer of this compound was confirmed by single crystal X-ray diffraction (see above) of crystals grown from $CH_2Cl_2$-hexane.*

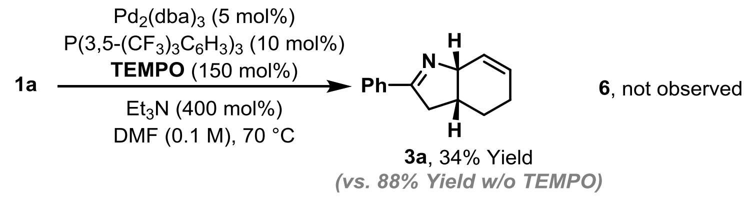


**(3a*R**,7a*S**)-2-Phenyl-3a,4,5,7a-tetrahydro-3*H*-indole 3a:** An oven-dried reaction tube, fitted with magnetic stirrer, was charged with $Pd_2(dba)_3$ (6.8 mg, 5 mol%), $P(3,5\text{-}(CF_3)_2C_6H_3)_3$ (20.0 mg, 20 mol%), oxime ester **1a** (61 mg, 0.15 mmol, 100 mol%) and TEMPO (36 mg, 0.23 mmol, 150 mol%). The tube was fitted with a rubber septum and purged with argon. DMF (1.50 mL, 0.1 M) and $Et_3N$ (84 μL, 400 mol%) were then added sequentially *via* syringe. The mixture was placed in a preheated heating block (70 °C) and stirred for 22 hours. The mixture was then cooled to room temperature and concentrated *in vacuo* (40 °C, approximately 1.0 mmHg). The residue was purified by FCC (8:1 hexane-EtOAc) to afford dihydropyrrole **3a** (12 mg, 41%, >19:1 d.r.) as a colorless oil. Oxime ester **1a** was recovered in 34% yield. Compound **6** was not detected. *Spectroscopic data for dihydropyrrole* ***3a*** *have been reported previously.*[1]

## Cyclopropane mechanistic experiments

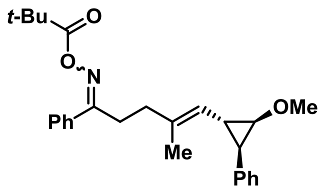


**(*E*)-5-(1*S**,2*R**,3*R**)-2-Methoxy-3-phenylcyclopropyl)-4-methyl-1-phenylpent-4-en-1-one *O*-pivaloyl oxime 7a:** This was synthesised according to our previously reported route.[2]

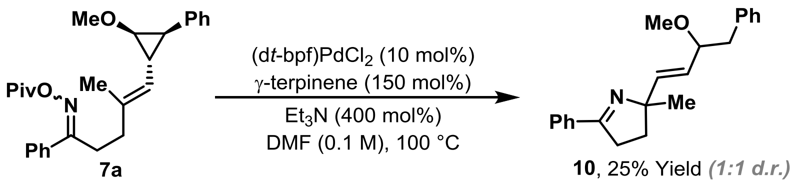


**(*E*)-2-(4-Methoxy-3-phenylbut-1-en-1-yl)-5-phenyl-3,4-dihydro-2*H*-pyrrole 10:** A flame-dried reaction tube, fitted with a magnetic stirrer bar, was charged with [d*t*-bpf]$PdCl_2$ (15.6 mg, 10 mol%) and oxime ester **7a** (100 mg, 0.239 mmol). The tube was fitted with a rubber septum and purged with argon. γ-Terpinene (153 μL, 0.955 mmol), $Et_3N$ (133 μL, 0.955 mmol) and argon-sparged DMF (2.4 mL) were added sequentially *via* syringe and the reaction mixture was heated at 90 °C. Upon consumption of starting material (TLC analysis, approximately 5 hours) the reaction mixture was cooled

to room temperature and concentrated *in vacuo* (approximately 0.1 mmHg). Purification of the residue by FCC (10:1 – 3:1 hexane-EtOAc) afforded the title compound **10** (19 mg, 25%, 1:1 d.r.) as a yellow oil. $\upsilon_{max}$ / $cm^{-1}$ (film) 1722, 1612, 1575, 1495; $\delta_H$ (400 MHz, $CDCl_3$) 7.87-7.83 (2H, m), 7.44-7.38 (3H, m), 7.25-7.13 (5H, m), 5.74 (1H, dd, *J* = 16.0, 2.5 Hz), 5.43-5.34 (1H, m), 3.79-3.73 (1H, m,), 3.24 (3H, d, *J* = 2.0 Hz), 3.05-2.69 (4H, m), 2.01-1.79 (2H, m), 1.40 (3H, app. d, *J* = 8.0 Hz); $\delta_C$ (100 MHz, $CDCl_3$) 171.4, 140.1, 139.7, 138.4, 134.6, 130.4, 129.6, 128.4, 128.0, 127.7, 126.5, 126.0, 83.2, 83.1, 75.9, 56.3, 56.2, 42.3, 35.8, 35.7, 34.8, 27.5, 27.4; HRMS (ESI$^+$) Found $[M+H]^+$: 320.2022, $C_{22}H_{26}NO$ requires 320.2009.

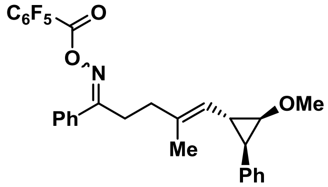


**(4*E*)-5-((1*S**,2*R**,3*R**)-2-Methoxy-3-phenylcyclopropyl)-4-methyl-1-phenylpent-4-en-1-one *O*-perfluorobenzoyl oxime 7b:** To a solution of (4*E*)-5-((1*S**,2*R**,3*R**)-2-methoxy-3-phenylcyclopropyl)-4-methyl-1-phenylpent-4-en-1-one oxime[2] (1.37 g, 3.95 mmol) and $Et_3N$ (1.38 mL, 7.90 mmol) in $CH_2Cl_2$ (12 mL) at 0 °C was added pentafluorobenzoyl chloride (0.68 mL, 4.74 mmol) dropwise over 5 minutes. The mixture was then warmed to room temperature and stirred until the reaction was complete as observed by TLC. MeOH (1 mL) was then added and the mixture stirred for a further 10 minutes. The mixture was diluted with $CH_2Cl_2$ (20 mL), washed with aq. 1 M HCl (15 mL), saturated aq. $Na_2CO_3$ (15 mL), brine (15 mL), dried ($Na_2SO_4$) and concentrated *in vacuo*. Purification of the residue by FCC (20:1 – 10:1 hexane-EtOAc) afforded the title compound **7b** (1.74 g, 83%, 12.5:1 mixture of oxime isomers) as a pale yellow oil. $\upsilon_{max}$ / $cm^{-1}$ (film) 1764, 1649, 1523, 1494, 1321, 1189, 1000; $\delta_H$ (400 MHz, $CDCl_3$) 7.74-7.71 (1.80H, m), 7.51-7.41 (3H, m), 7.32-7.16 (5.2H, m), 4.79-4.73 (1H, m), 3.36 (0.08H, dd, *J* = 6.5, 3.5 Hz), 3.33 (0.92H, m, dd, *J* = 6.5, 3.5 Hz), 3.18 (0.24H, s), 3.17 (2.76H, s), 3.06-2.95 (1.84H, m), 2.89-2.85 (0.16H, m), 2.29-2.19 (2H, m), 2.01 (1H, ddd, *J* = 8.5, 6.5, 3.5 Hz), 1.91 (1H, app. t, *J* = 6.5 Hz), 1.73 (3H, s); $\delta_C$ (100 MHz, $CDCl_3$) *Signals for the major isomer only:* 168.5, 156.6, 137.1, 134.3, 133.2, 131.0, 128.8, 128.0, 127.7, 127.4, 125.7, 67.1, 58.2, 36.0, 32.3, 27.7, 26.5, 16.5; -136.9 (2F), -137.3 (0.16F), -147.4 (1F), -148.1 (0.08F), -159.8 (2F), -160.2 (0.16F); HRMS (ESI$^+$) Found $[M+H]^+$: 530.1747, $C_{29}H_{25}F_5NO_3$ requires 530.1749.

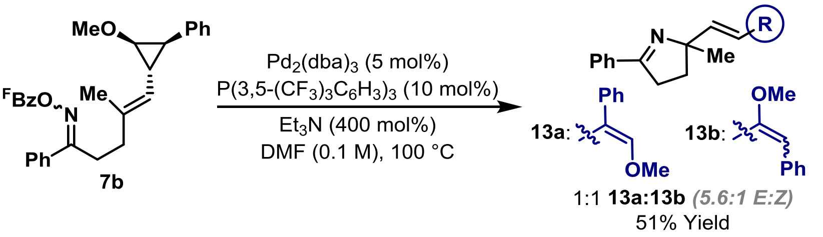

**2-((1*E*,3*E*)-4-Methoxy-3-phenylbuta-1,3-dien-1-yl)-2-methyl-5-phenyl-3,4-dihydro-2*H*-pyrrole 13a *and* 2-((1*E*)-3-Methoxy-4-phenylbuta-1,3-dien-1-yl)-2-methyl-5-phenyl-3,4-dihydro-2*H*-pyrrole 13b:** A flame-dried reaction tube, fitted with a magnetic stirrer bar, was charged with oxime ester **7b** (150 mg, 0.284 mmol), $Pd_2(dba)_3$ (6.5 mg, 7.1 µmol) and $P(3,5\text{-}(CF_3)_2C_6H_3)_3$ (19 mg, 0.0284 mmol). The tube was fitted with a rubber septum and purged with argon. $Et_3N$ (158 µL, 1.13 mmol) and argon-sparged DMF (2.8 mL) were added sequentially *via* syringe and the reaction mixture was heated at 90 °C. Upon consumption of starting material (TLC analysis) the reaction mixture was cooled to room temperature and concentrated *in vacuo* (approximately 0.1 mmHg). Purification of the residue by FCC (50:1 – 30:1 toluene-EtOAc) afforded an inseparable mixture of the title compounds (46 mg, 51% yield, 1:1, **13a**:**13b**) as a yellow oil. **13b** was formed as a 5.6:1 mixture of *E*:*Z* alkene isomers. *Due to product instability, characterization was only possible by* $^{1}$*H and* $^{13}$*C NMR, and assignments were made by 2D NMR techniques (see pages S20-21).* $\delta_H$ (400 MHz, $CDCl_3$) 7.88-7.79 (2H, m), 7.44-7.10 (8H, m), 6.74 (0.50H, dd, *J* = 16.5, 1.0 Hz), 6.49 (0.43H, d, *J* = 15.5 Hz), 6.46 (0.43H, d, *J* = 15.5 Hz), 6.25 (0.07H, d, *J* = 15.5 Hz), 6.01 (0.07H, *J* = 15.5 Hz), 6.00 (0.5H, m), 5.82-5.74 (1H, m), 3.72 (1.27H, s), 3.69 (1.5H, s), 3.63 (0.21H, s), 3.08-2.86 (2H, m), 2.12-2.04 (1H, m), 1.93-1.86 (1H, m), 1.50 (0.21H, s), 1.46 (1.27H, s), 1.43 (1.5H, s); $\delta_C$ (100 MHz, $CDCl_3$) 171.6 (2C), 170.8 (3C), 155.5, 153.9, 146.6, 138.3, 138.1, 137.4, 136.8, 136.8, 135.7, 134.8, 134.6 (2C), 130.5, 130.4, 130.2, 129.5, 129.2, 128.6, 128.4, 128.3 (3C), 128.2, 128.1, 128.0, 127.8 (2C), 126.6, 126.5, 125.5, 123.3, 120.9, 119.9, 119.8, 116.3, 103.2, 76.8, 76.2, 76.2, 60.2, 58.5, 54.8, 36.2, 36.0, 35.9, 35.0 (2C), 34.9, 27.6, 27.5, 27.2.

*The relative stereochemistry of* ***(E)-13b*** *and* ***(Z)-13b*** *was determined by nOe analysis, as indicated on the compound structures below.*

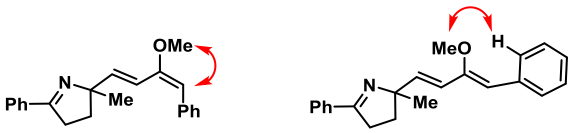


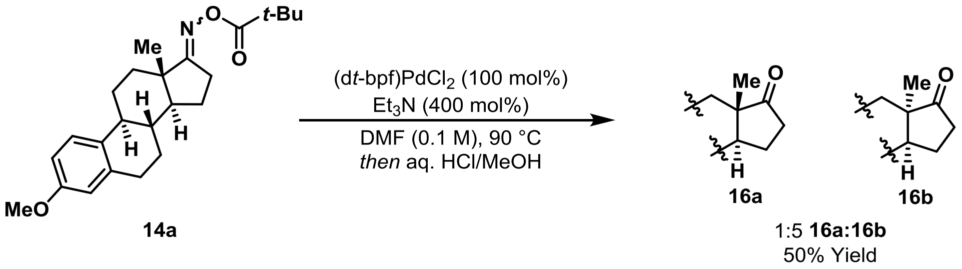


**Estrone-3-methylether 16a *and* 13α-estrone-3-methylether 16b:** An oven-dried reaction tube, fitted with magnetic stirrer, was charged with (d*t*-bpf)$PdCl_2$ (51 mg, 0.078 mmol) and oxime ester **14a**[2] (30 mg, 0.078 mmol). The tube was fitted with a rubber septum and purged with argon. $Et_3N$ (22 µL, 0.156 mmol) and DMF (0.8 mL) were then added sequentially *via* syringe. The mixture was then placed in a

preheated heating block (90 °C) and stirred for 18 hours. The mixture was then cooled to room temperature and concentrated *in vacuo* (40 °C, approximately 1.0 mmHg). The residue was re-dissolved in MeOH (4 mL) and 1 M HCl (2 mL) was added. The mixture was heated at 75 °C in a sealed tube for 2 hours. After cooling to room temperature the mixture was diluted with $Et_2O$ (10 mL) and washed with $Na_2CO_3$ (10 mL), brine (10 mL), dried ($Na_2SO_4$), filtered and concentrated *in vacuo*. Purification of the residue (5:1 hexane-EtOAc) afforded the title compounds as an inseparable mixture (11 mg, 50% yield, 1:5, **16a**:**16b**). This material was contaminated with approx. 5% of an unidentified impurity. $\delta_H$ (400 MHz, $CDCl_3$) Diagnostic signals for **16a**: 0.91 (0.16H, s); Diagnostic signals for **16b**: 1.06 (0.84H, s); $\delta_C$ (100 MHz, $CDCl_3$) Diagnostic signals for **16a**: 21.6, 13.8; Diagnostic signals for **16b**: 21.0. *The spectroscopic properties of these compounds were consistent with the data previously reported in the literature.*[3] *The most diagnostic method for characterizing the mixture was* $^{13}$*C NMR (shown below).*

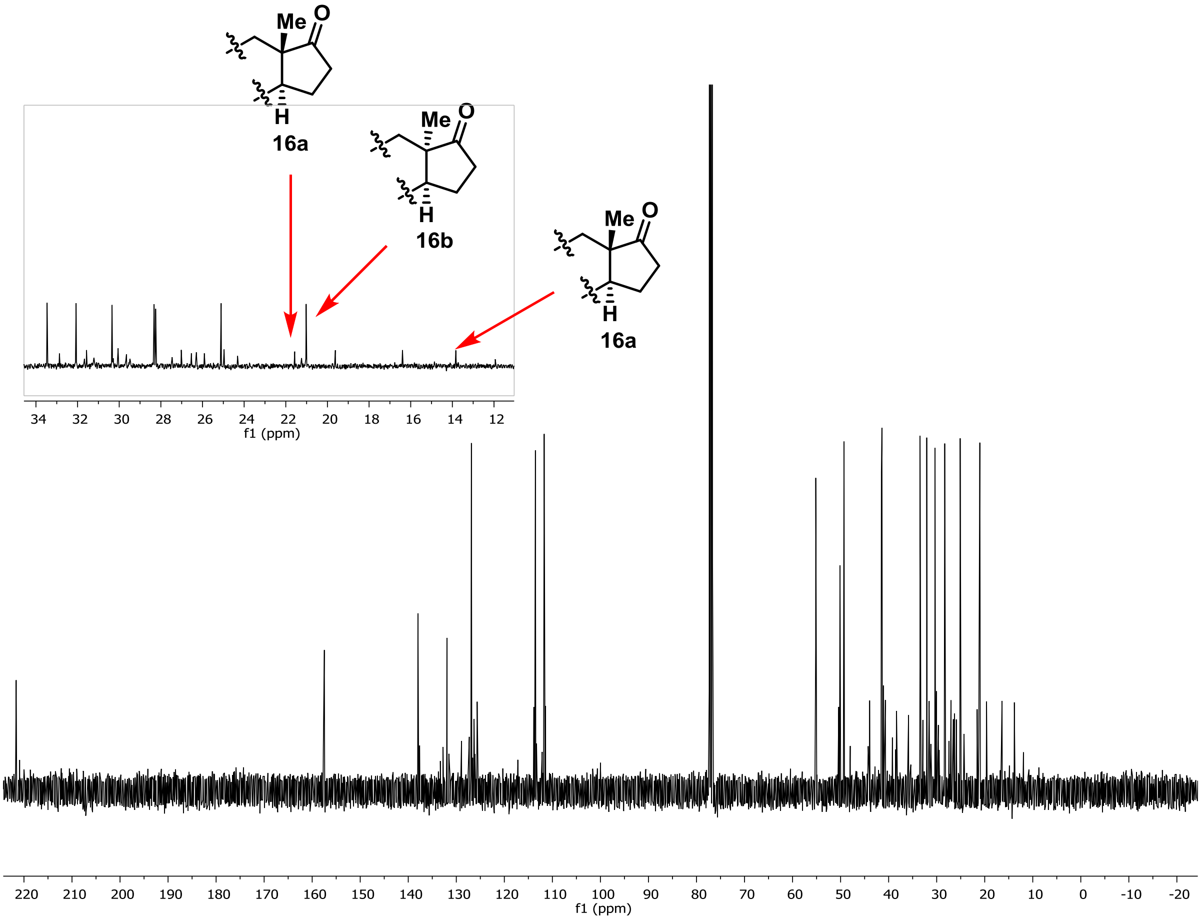

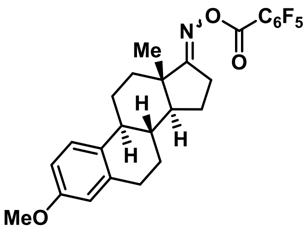


**Estrone 3-methyl ether *O*-pentafluorobenzoyl oxime 14b:** To a solution of estrone-3-methylether oxime[2] (500 mg, 1.67 mmol) and $Et_3N$ (0.58 mL, 3.34 mmol) in $CH_2Cl_2$ (5 mL) at 0 °C was added pentafluorobenzoyl chloride (0.29 mL, 2.00 mmol) dropwise over 5 minutes. The mixture was then warmed to room temperature and stirred until the reaction was complete as observed by TLC. MeOH (1 mL) was then added and the mixture stirred for a further 10 minutes. The mixture was diluted with $CH_2Cl_2$ (10 mL), washed with 1 M HCl (10 mL), saturated aq. $Na_2CO_3$ (10 mL), brine (10 mL), dried ($Na_2SO_4$) and concentrated *in vacuo*. Purification of the residue by FCC (10:1 hexane-EtOAc) afforded the title compound **14b** (552 mg, 67%, >19:1 mixture of oxime isomers) as a colorless waxy solid. $\upsilon_{max}$ / $cm^{-1}$ (film) 1756, 1612, 1523, 1494, 1321, 1189, 1000; $\delta_H$ (400 MHz, $CDCl_3$) 7.22 (1H, dd, *J* = 8.5, 1.0 Hz), 6.73 (1H, dd, *J* = 8.5, 3.0 Hz), 6.64 (1H, d, *J* = 3.0 Hz), 3.78 (3H, s), 2.92-2.87 (2H, m), 2.80-2.60 (2H, m), 2.46-2.40 (1H, m), 2.35-2.23 (2H, m), 1.99-1.93 (2H, m), 1.80 (1H, td, *J* = 13.5, 4.0 Hz), 1.63-1.40 (5H, m), 1.08 (3H, s); $\delta_C$ (100 MHz, $CDCl_3$) 180.8, 157.6, 137.6, 131.9, 126.3, 113.9, 111.6, 55.2, 52.9, 46.0, 43.7, 38.2, 33.6, 29.6, 27.8, 27.2, 26.0, 22.8, 17.1; $\delta_F$ (376 MHz, $CDCl_3$) -137.0 (2F), -147.4 (1F), -159.7 (2F); HRMS (ESI$^+$) Found $[M+Na]^+$: 516.1555, $C_{26}H_{24}F_5NO_3Na$ requires 516.1569.

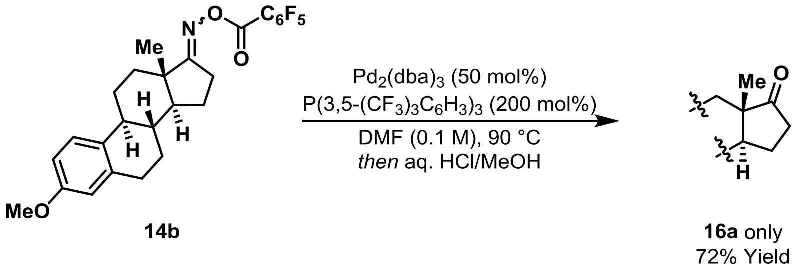


**Estrone-3-methylether 16a:** An oven-dried reaction tube, fitted with magnetic stirrer, was charged with $Pd_2(dba)_3$ (42 mg, 0.046 mmol), $P(3,5-(CF_3)_2C_6H_3)_3$ (122 mg, 0.182 mmol) and oxime ester **14b** (45 mg, 0.091 mmol). The tube was fitted with a rubber septum and purged with argon. Anhydrous DMF (1 mL) was then added *via* syringe. The mixture was then placed in a preheated heating block (90 $^oC$) until complete consumption of starting material was observed (TLC analysis). The mixture was then cooled to room temperature and concentrated *in vacuo* (40 °C, approximately 1.0 mmHg). The residue was redissolved in MeOH (4 mL) and 1 M HCl (2 mL) was added. The mixture was heated at 75 °C in a sealed tube for 2 hours. After cooling to room temperature, the mixture was diluted with $Et_2O$ (20 mL) and washed with $NaHCO_3$ (10 mL), brine (10 mL), dried ($Na_2SO_4$), filtered and concentrated *in vacuo*. Purification of the residue (10:1 – 5:1 hexane-EtOAc) afforded the title

compound **16a** (19 mg, 72%) as a colorless solid. The product was contaminated with approx. 2 mg of dibenzylideneacetone; the yield of **16a** has been adjusted accordingly. $\delta_H$ (400 MHz, $CDCl_3$) 7.21 (1H, d, $J$ = 8.5 Hz), 6.73 (1H, dd, $J$ = 8.5, 3.0 Hz), 6.65 (1H, d, $J$ = 3.0 Hz), 3.78 (3H, s), 2.94-2.88 (2H, m), 2.54-2.47 (1H, m), 2.43-2.38 (1H, m), 2.29-2.23 (1H, m), 2.19-1.94 (4H, m), 1.68-1.39 (6H, m), 0.91 (3H, s); $\delta_C$ (100 MHz, $CDCl_3$) 220.9, 157.4, 137.7, 132.0 126.3, 113.9, 111.5, 55.2, 50.4, 48.0, 44.0, 38.4, 35.9, 31.6, 29.7, 26.5, 25.9, 21.6, 13.8. *The spectroscopic properties of this compound were consistent with the available in the literature.*[3] ***16b** was not observed in this reaction, see the $^{13}C$ NMR spectrum below.*

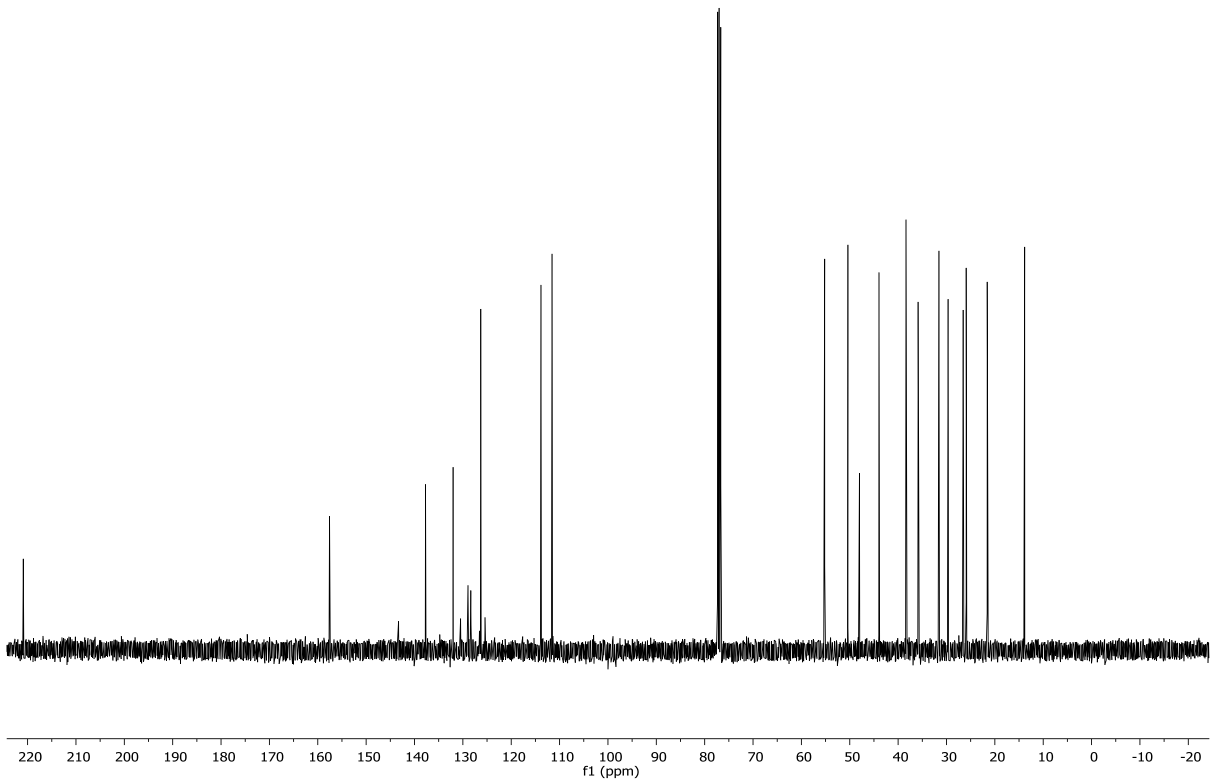


## Iminyl radical cyclization scope

**<u>General Procedure A</u>: for 'reductive' aza-Heck cyclizations:** An oven-dried reaction tube, fitted with magnetic stirrer, was charged with (d*t*-bpf)$PdCl_2$ (5 mol%) and oxime ester substrate (100 mol%). The tube was fitted with a rubber septum and purged with argon. DMF (0.1 M w.r.t oxime ester), $Et_3N$ (400 mol%) and γ-terpinene (400 mol%) were then added sequentially *via* syringe. The mixture was placed in a preheated heating block (70-95 °C as noted) and stirred for 20-24 hours (as noted). The mixture was then cooled to room temperature and concentrated *in vacuo* (40 °C, approximately 1.0 mmHg). The residue was purified by FCC, under the conditions noted, to afford the target dihydropyrrole.

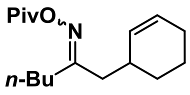


**1-(Cyclohex-2-en-1-yl)hexan-2-one *O*-pivaloyl oxime 21a:** To a room temperature solution of 1-(cyclohex-2-en-1-yl)hexan-2-one oxime (515 mg, 2.64 mmol)[1] in anhydrous $CH_2Cl_2$ (7.80 mL) was added pivaloyl chloride (0.40 mL, 3.21 mmol) and $Et_3N$ (0.73 mL, 5.25 mmol). The mixture was stirred at room temperature for 22 hours. MeOH (5 mL) was added and the mixture was diluted with $Et_2O$ (50 mL). The mixture was washed with saturated aq. $NaHCO_3$ (2 × 25 mL), dried ($Na_2SO_4$) and concentrated *in vacuo*. Purification of the residue by FCC (6:1 hexane-EtOAc) afforded oxime ester **21a** (688 mg, 96%, 1:1 mixture of isomers) as a colorless oil. $\upsilon_{max}$ / $cm^{-1}$ (film) 2932, 1755, 1702, 1114;$\delta_H$ (400 MHz, $CDCl_3$) 5.77-5.70 (2H, m), 5.57 (1H, m), 5.50 (1H, m), 2.55-2.25 (10H, m), 2.03-1.95 (4H, m), 1.85-1.68 (4H, m), 1.63-1.45 (6H, m), 1.45-1.25 (6H, m), 1.29 (9H, s), 1.27 (9H, s), 0.97-0.90 (6H, m); $\delta_C$ (100 MHz, $CDCl_3$) 175.4, 175.3, 169.5, 169.3, 130.2, 130.1, 128.4, 128.3, 40.6, 38.8 (2 signals), 35.5, 34.5, 33.0, 32.6, 29.6, 29.3, 29.0, 28.8, 28.3, 27.4, 26.6, 25.2, 25.1, 23.1, 22.7, 21.2, 21.1, 13.9 (2 signals); HRMS ($ESI^+$) Found $[M+H]^+$: 280.2274, $C_{17}H_{30}NO_2$ requires 280.2271.

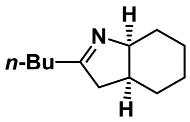


**(3*aS**,7*aS**)-2-Butyl-3*a*,4,5,6,7,7*a*-hexahydro-3*H*-indole 22a:** General procedure A: Oxime ester **21a** (50 mg, 0.18 mmol) was employed. The reaction was heated at 95 °C for 24 hours. FCC (2:1 to 1:1 hexane-EtOAc) afforded the title compound **22a** (15 mg, 47%) as a yellow oil. $\upsilon_{max}$ / $cm^{-1}$ (film) 2927, 1633, 1450, 1295; $\delta_H$ (400 MHz, $CDCl_3$) 3.75 (1H, m), 2.46 (1H, ddd, *J* = 17.0, 8.0, 2.0 Hz), 2.35 (2H, t, *J* = 8.0 Hz), 2.25-2.17 (2H, m), 1.89-1.73 (2H, m), 1.60-1.50 (2H, m), 1.50-1.09 (8H, m), 0.93 (3H, t, *J* = 7.5 Hz); $\delta_C$ (100 MHz, $CDCl_3$) 179.2, 69.0, 43.8, 36.9, 34.4, 29.0, 28.7, 27.6, 23.2, 22.8, 21.9, 14.0; HRMS ($EI^+$) Found $[M]^+$: 289.1838, $C_{21}H_{23}N$ requires 289.1830. *N.B. Compound **22a** is relatively volatile and appropriate care must be taken.*

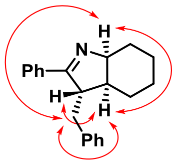


**(3*S**,3*aS**,7*aS**)-3-Benzyl-2-phenyl-3*a*,4,5,6,7,7*a*-hexahydro-3*H*-indole 22b:** General procedure A: Oxime ester **21b** (71 mg, 0.18 mmol)[2] was employed. The reaction was heated at 80 °C for 22 hours. FCC (5:1 hexane-EtOAc) afforded the title compound **22b** (38 mg, 73%, >19:1 d.r.) as a colorless oil.$\upsilon_{max}$ / $cm^{-1}$ (film) 2925, 1603, 1572, 1495, 1447; $\delta_H$ (400 MHz, $CDCl_3$) 7.97-7.90 (2H, m), 7.49-7.44 (3H, m), 7.35-7.30 (2H, m), 7.27-7.20 (3H, m), 4.05 (1H, m), 3.30 (1H, dd, *J* = 10.5, 4.0 Hz), 2.91 (1H, dd, *J* = 14.5, 4.0 Hz), 2.65 (1H, dd, *J* = 14.5, 11.0 Hz), 2.40 (1H, m), 2.11 (1H, dddd, *J* = 5.5, 5.5, 5.5, 4.0 Hz), 1.82-1.71 (1H, m), 1.60-1.47 (3H, m), 1.31 (1H, m), 1.15 (1H, m), 1.02 (1H, m); $\delta_C$ (100 MHz,

CDCl$_3$) 176.0, 140.5, 134.3, 130.4, 128.8, 128.7, 128.6, 128.0, 126.3, 67.7, 57.3, 41.1, 34.7, 29.1, 28.7, 24.0, 21.8. HRMS (EI$^+$) Found [M]$^+$: 289.1838, $C_{21}H_{23}N$ requires 289.1830.

*The relative stereochemistry of this compound was determined by nOe analysis, as indicated on the compound structure.*

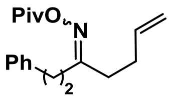


**1-Phenylhept-6-en-3-one *O*-pivaloyl oxime 21c:** To a solution of the corresponding oxime[4] (500 mg, 2.46 mmol) and pivaloyl chloride (593 mg, 4.92 mmol) in $CH_2Cl_2$ (25 mL) was added triethylamine (1.13 mL, 8.12 mmol) at r.t.. After 20 hours the mixture was diluted with water (10 mL) and extracted with $CH_2Cl_2$ (3 × 10 mL). The organic extracts were combined, dried ($Na_2SO_4$) and concentrated *in vacuo*. The residue purified by FCC (EtOAc-hexane 1:1) to afford **21c** (619 mg, 88 %, 1:1 d.r. *A*:*B*) as a colorless oil. $\upsilon_{max}$ / cm$^{-1}$ (film) 2933, 1754, 1107, 1027, 912, 699; $\delta_H$ (400 MHz, CDCl$_3$) 7.33-7.18 (5H, *A and B*), 5.87-5.74 (1H, *A and B*), 5.09-4.99 (2H, *A and B*), 2.95-2.91 (2H, m, *A*), 2.87-2.83 (2H, m, *B*), 2.68-2.63 (2H, m, *A and B*), 2.51-2.47 (2H, m, *A*), 2.41-2.26 (4H, m, *A and B*), 1.29 (9H, s, *A and B*); $\delta_C$ (100 MHz, CDCl$_3$) 175.2 (*A and B*), 168.9 (*A*), 168.7 (*B*), 140.9 (*A*), 140.3 (*B*), 136.9 (*A*), 136.6 (*B*), 128.8, 128.7, 128.4 and 128.3 (*A and B*), 126.7 (*A*), 126.4 (*B*), 116.1 (*A*), 115.8 (*B*), 38.9 (*A*), 38.8 (*B*), 36.6, 34.2, 32.5 and 32.2, (*A and B*), 31.9, 30.3, 30.2 and 29.5 (*A and B*), 27.4 (*A and B*); HRMS (ESI$^+$) Found: [M+Na]$^+$ 310.1777, $C_{18}H_{25}NO_2Na$ requires 310.1783.

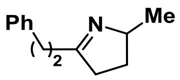


**2-Methyl-5-phenethyl-3,4-dihydro-2*H*-pyrrole 22c:** General procedure A: Oxime ester **21c** (53 mg, 0.18 mmol) was employed. The reaction was heated at 80 °C for 24 hours. FCC (EtOAc) afforded the title compound **22c** (19 mg, 58%) as a pale brown oil. $\delta_H$ (400 MHz, CDCl$_3$) 7.33-7.28 (2H, m), 7.26-7.19 (3H, m), 4.12-4.02 (1H, m), 3.02-2.88 (2H, m), 2.68-2.62 (2H, m), 2.55 (1H, dddd, $J$ = 17.0, 10.0, 5.0, 2.0 Hz), 2.49-2.39 (1H, m), 2.14-2.05 (1H, m), 1.44-1.34 (1H, m), 1.27 (3H, d, $J$ = 7.0 Hz); $\delta_C$ (100 MHz, CDCl$_3$) 176.3, 141.6, 128.5, 128.4, 126.1, 67.8, 37.9, 35.6, 32.9, 30.8, 22.2.

*The spectroscopic properties of this compound were consistent with the data available in the literature.*[5]

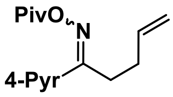


**1-(Pyridin-4-yl)pent-4-en-1-one *O*-pivaloyl oxime 21d:** In a re-sealable tube, 1-(pyridin-4-yl)pent-4-en-1-one[6] (499 mg, 3.10 mmol), $NH_2OH·HCl$ (253 mg, 3.64 mmol) and NaOAc (298 mg, 3.63 mmol) were suspended in MeOH (9.00 mL). The tube was sealed and the mixture was heated at 80 °C for 16 hours. The mixture was cooled to room temperature and concentrated *in vacuo*. The residue was dissolved in EtOAc (50 mL), washed with saturated aq. $NaHCO_3$ (50 mL) and brine (50 mL), dried

($Na_2SO_4$) and concentrated *in vacuo* to afford the corresponding oxime (515 mg, 94%) as a pale yellow solid, which was used directly without purification. To a room temperature solution of the oxime (515 mg, 2.91 mmol)[1] in anhydrous $CH_2Cl_2$ (9.00 mL) was added pivaloyl chloride (0.42 mL, 3.41 mmol) and $Et_3N$ (0.81 mL, 5.82 mmol). The mixture was stirred at room temperature for 22 hours. MeOH (5 mL) was added and the mixture was concentrated *in vacuo*. The residue was diluted with EtOAc (50 mL), and washed sequentially with water (50 mL), saturated aq. $NaHCO_3$ (2 × 25 mL) and brine (50 mL). The organic portion was dried ($Na_2SO_4$) and concentrated *in vacuo*. Purification of the residue by FCC (1:3 hexane-EtOAc) afforded oxime ester **21d** (696 mg, 92%, 10:1 mixture of isomers) as a pale yellow oil. $\upsilon_{max}$ / $cm^{-1}$ (film); 2978, 1699, 1482, 1197; $\delta_H$ (400 MHz, $CDCl_3$) *Signals for the major isomer:* 8.69-8.67 (2H, m), 7.62-7.60 (2H, m), 5.79 (1H, ddt, *J* = 17.0, 10.0, 6.5), 5.09-5.01 (2H, m), 2.94-2.89 (2H, m), 2.37-2.29 (2H, m), 1.33 (9H, s). *Characteristic signal for the minor isomer:* 2.77 (0.2H, t, *J* = 7.5); $\delta_C$ (100 MHz, $CDCl_3$) *Signals for the major isomer only:* 174.6, 164.0, 150.3, 141.6, 135.9, 121.3, 116.4, 38.8, 30.5, 27.4, 27.2; HRMS: ($ESI^+$) Found $[M+H]^+$ 261.1599, $C_{15}H_{21}N_2O_2$ requires 261.1598.

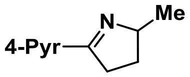


**4-(2-Methyl-3,4-dihydro-2*H*-pyrrol-5-yl)pyridine 22d:** General procedure A: Oxime ester **21d** (49 mg, 0.19 mmol) was employed. The reaction was heated at 90 °C for 24 hours. FCC (10:1 EtOAc-MeOH) afforded the title compound **22d** (25 mg, 82%) as a yellow oil. $\upsilon_{max}$ / $cm^{-1}$ (film) 2926, 1595, 1408; $\delta_H$ (400 MHz, $CDCl_3$) 8.66 (2H, d, *J* = 5.5), 7.66-7.65 (2H, m), 4.36-4.26 (1H, m), 3.02 (1H, dddd, *J* = 17.0, 10.0, 5.0, 2.5), 2.86 (1H, dddd, *J* = 17.0, 10.0, 7.5, 2.5), 2.27 (1H, dddd, *J* = 12.5, 10.0, 7.5, 5.0), 1.58 (1H, dddd, *J* = 12.5, 10.0, 7.5, 6.5), 1.36 (3H, d, *J* = 7.0); $\delta_C$ (100 MHz, $CDCl_3$) 170.3, 150.2, 141.6, 121.7, 69.0, 35.0, 30.5, 21.9; HRMS: ($CI^+$) Found $[M+H]^+$ 161.1077, $C_{10}H_{13}N_2$ requires 161.1079.

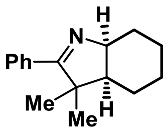


**(3*aS**,7*aS**)-3,3-Dimethyl-2-phenyl-3*a*,4,5,6,7,7*a*-hexahydro-3*H*-indole 22e:** General procedure A: Oxime ester **21e** (60 mg, 0.18 mmol)[2] was employed. The reaction was heated at 90 °C for 21 hours. FCC (4:1 hexane-EtOAc) afforded the title compound **22e** (29 mg, 71%) as a colorless oil. $\upsilon_{max}$ / $cm^{-1}$ (film) 2927, 1597, 1445, 1299; $\delta_H$ (400 MHz, $CDCl_3$) 7.55 (2H, m), 7.31-7.25 (3H, m), 4.01 (1H, ddd, *J* = 5.5, 5.5, 4.0), 2.17 (1H, dddd, *J* = 14.0, 4.0, 4.0, 4.0), 1.82 (1H, m), 1.73 (1H, m), 1.55-1.44 (3H, m), 1.35-1.25 (1H, m), 1.25 (3H, s), 1.15-1.07 (5H, m); $\delta_C$ (125 MHz, $CDCl_3$) 180.4, 135.9, 129.1, 128.0, 127.5, 65.9, 53.4, 49.7, 29.4, 24.3, 24.2, 24.0, 22.2, 21.2; HRMS: ($ESI^+$) Found $[M+H]^+$ 228.1751, $C_{16}H_{22}N$ requires 228.1747.

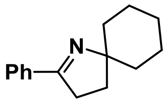

**2-Phenyl-1-azaspiro[4.5]dec-1-ene 22f:** General procedure A: Oxime ester **21f** (60 mg, 0.19 mmol)[2] was employed. The reaction was heated at 70 °C for 24 hours. FCC (12:1 hexane-EtOAc) afforded the title compound **22f** (32 mg, 80%) as a pale brown oil. $\upsilon_{max}$ / cm$^{-1}$ (film) 2926, 2853, 1616, 1447, 1335; $\delta_H$ (400 MHz, $CDCl_3$) 7.84 (2H, m), 7.41-7.38 (3H, m), 2.98 (2H, t, *J* = 8.0), 1.86 (2H, t, *J* = 8.0), 1.79 (4H, m), 1.50-1.38 (5H, m), 1.65 (1H, m); $\delta_C$ (100 MHz, $CDCl_3$) 169.5, 135.2, 130.2, 128.4, 127.8, 76.9, 37.8, 34.5, 32.8, 25.9, 23.7; HRMS: ($CI^+$) Found $[M+H]^+$ 214.1588, $C_{15}H_{19}N$ requires 214.1596.

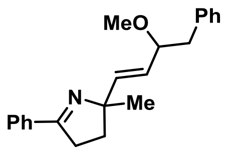
MeO
Ph
N
Me
Ph

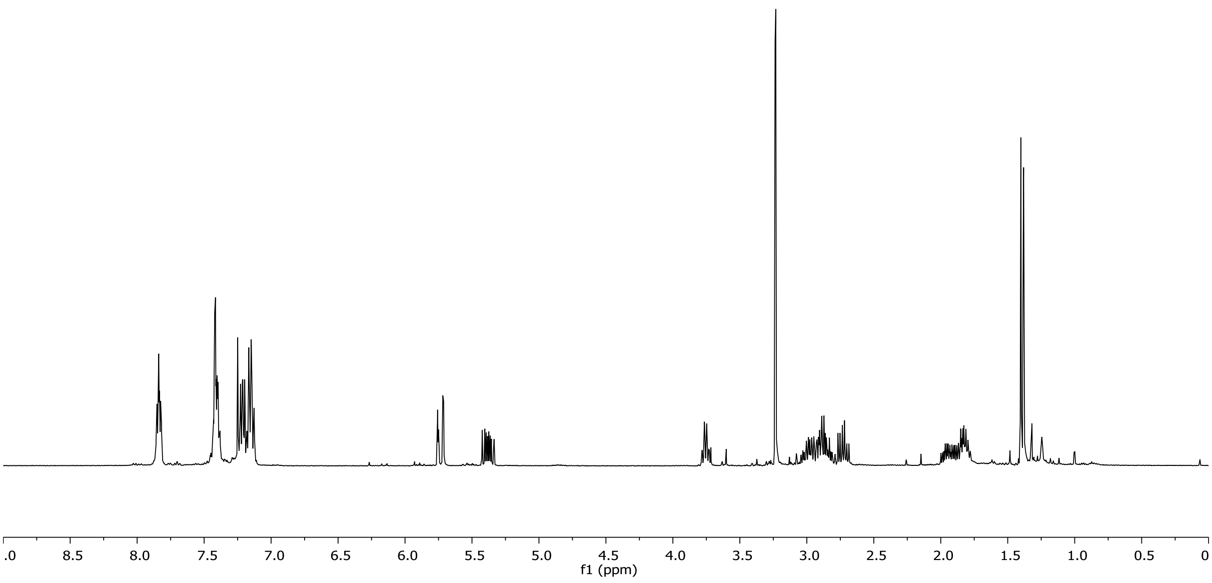
f1 (ppm)

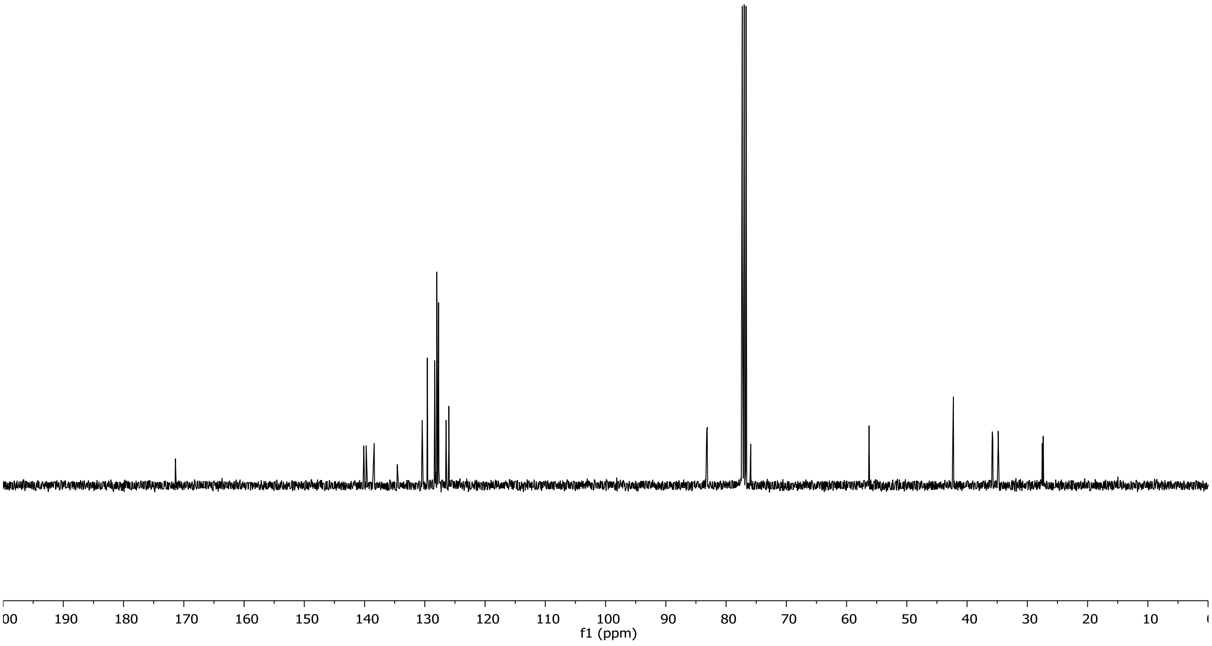
f1 (ppm)

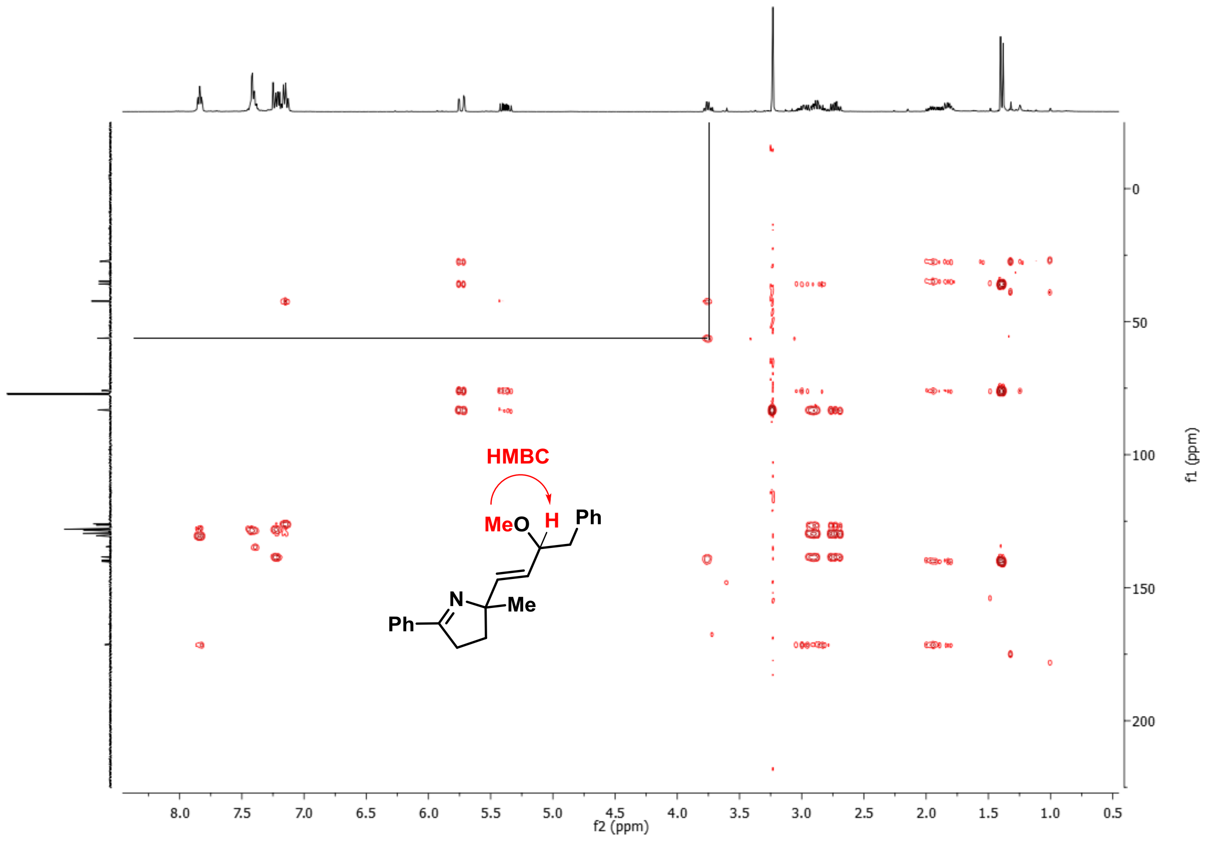
HMBC
MeO
H
Ph
N
Me
Ph
f2 (ppm)
f1 (ppm)

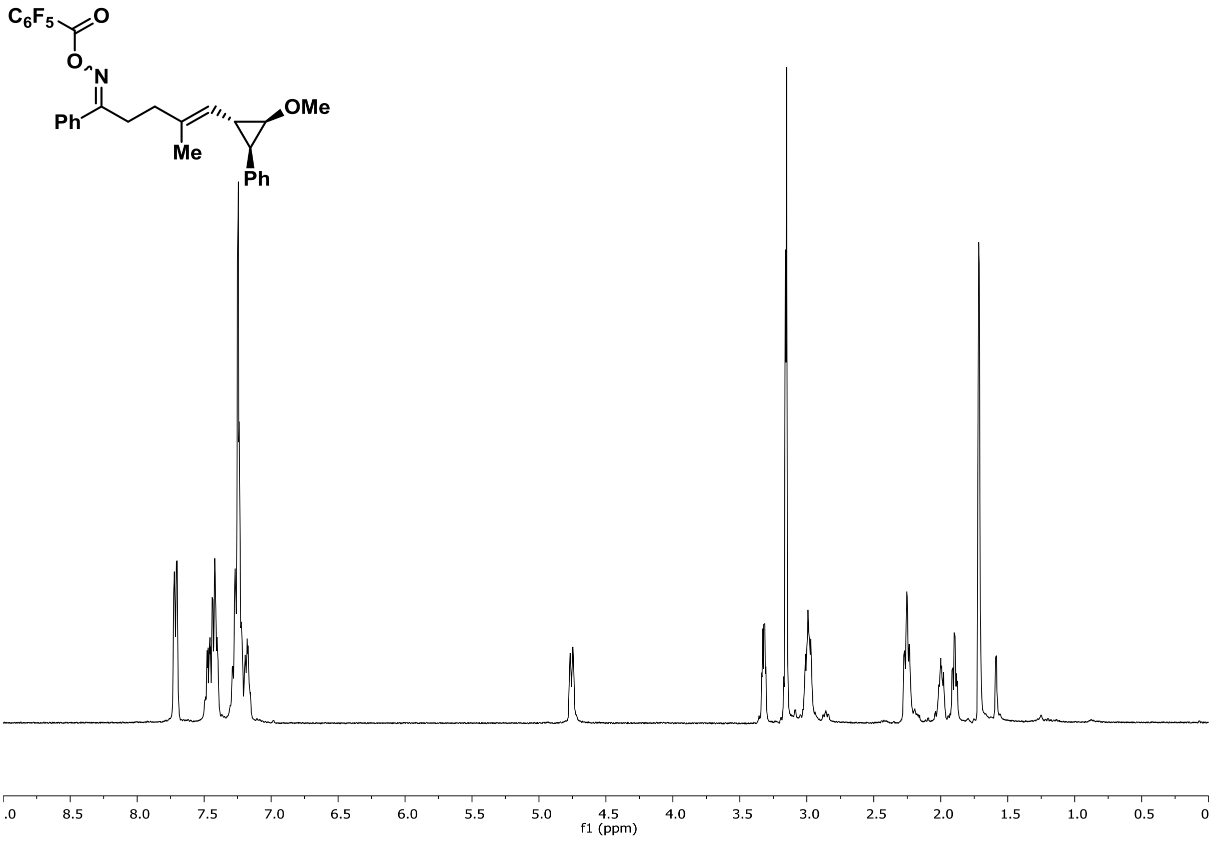
C6F5
O
O
N
Ph
Me
OMe
Ph
.0 8.5 8.0 7.5 7.0 6.5 6.0 5.5 5.0 4.5 4.0 3.5 3.0 2.5 2.0 1.5 1.0 0.5 0
f1 (ppm)

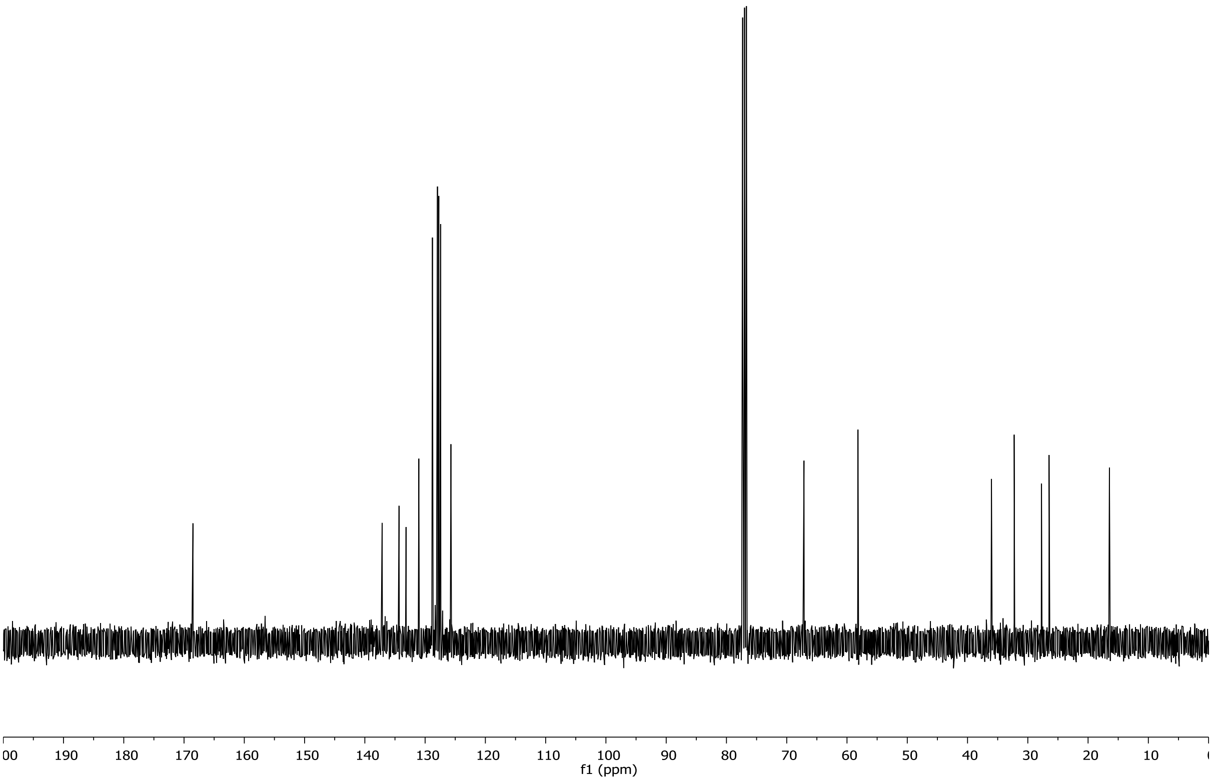
00 190 180 170 160 150 140 130 120 110 100 90 80 70 60 50 40 30 20 10
f1 (ppm)

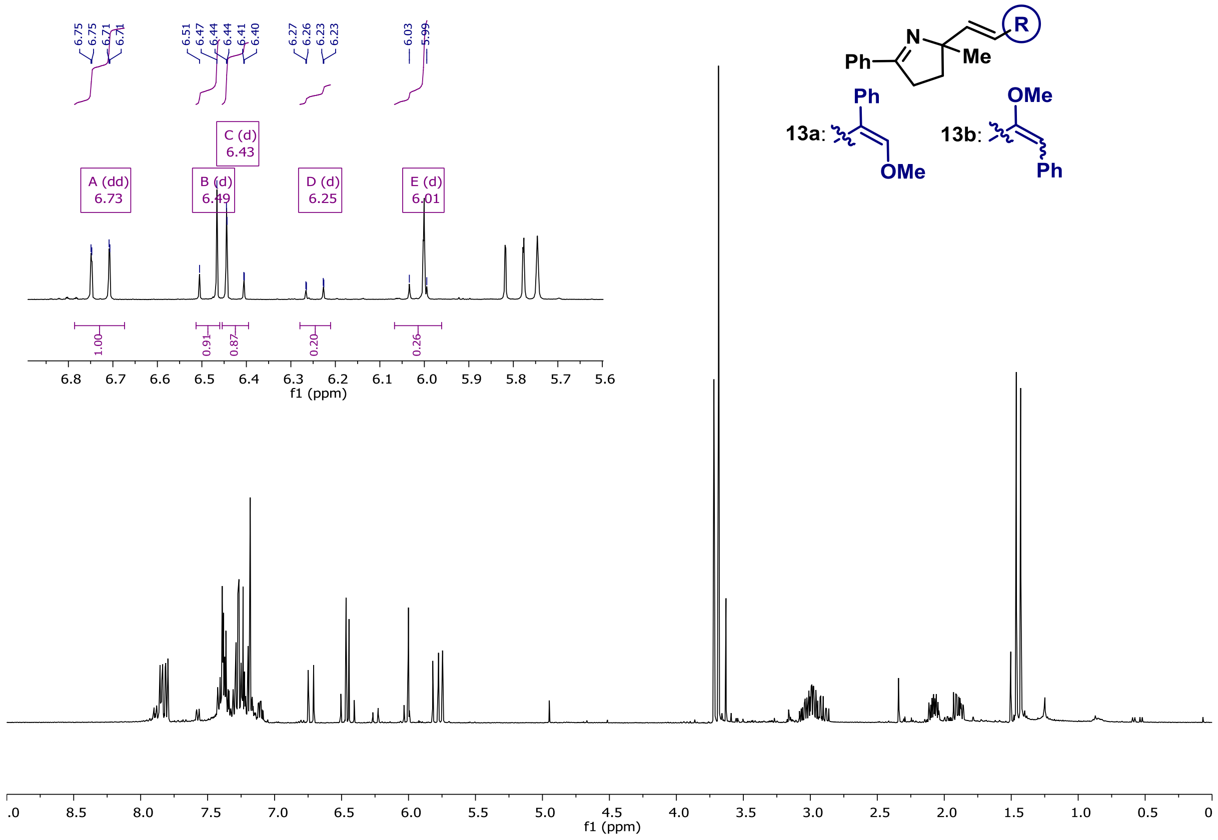

Ph
N
Me
R
13a:
Ph
OMe
13b:
OMe
Ph
A (dd) 6.73
B (d) 6.49
C (d) 6.43
D (d) 6.25
E (d) 6.01
1.00
0.91
0.87
0.20
0.26
f1 (ppm)
f1 (ppm)


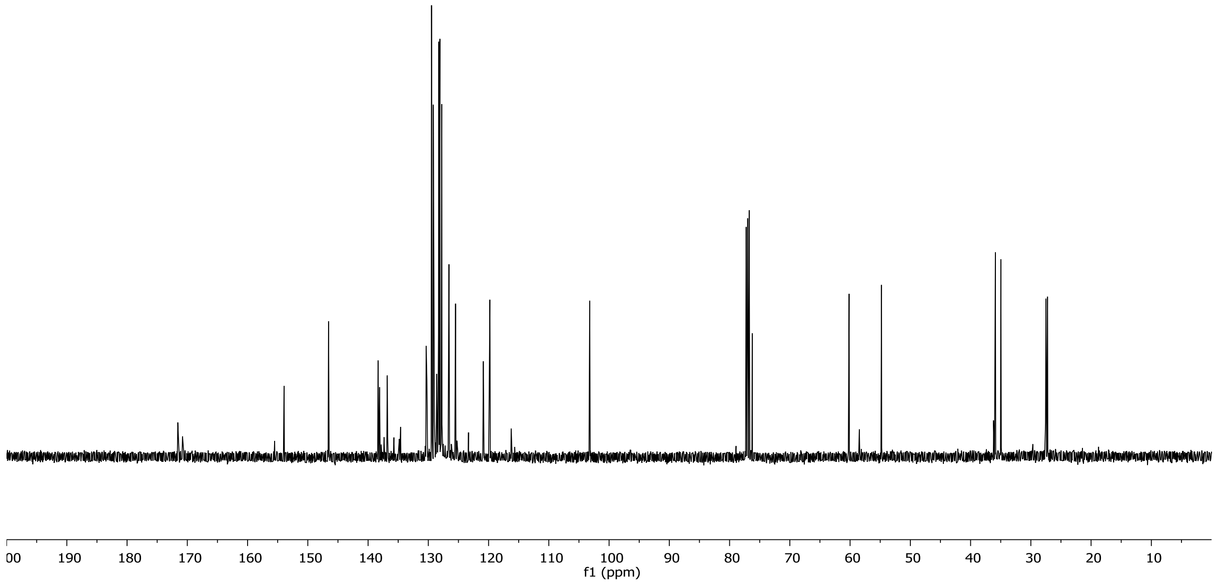

f1 (ppm)

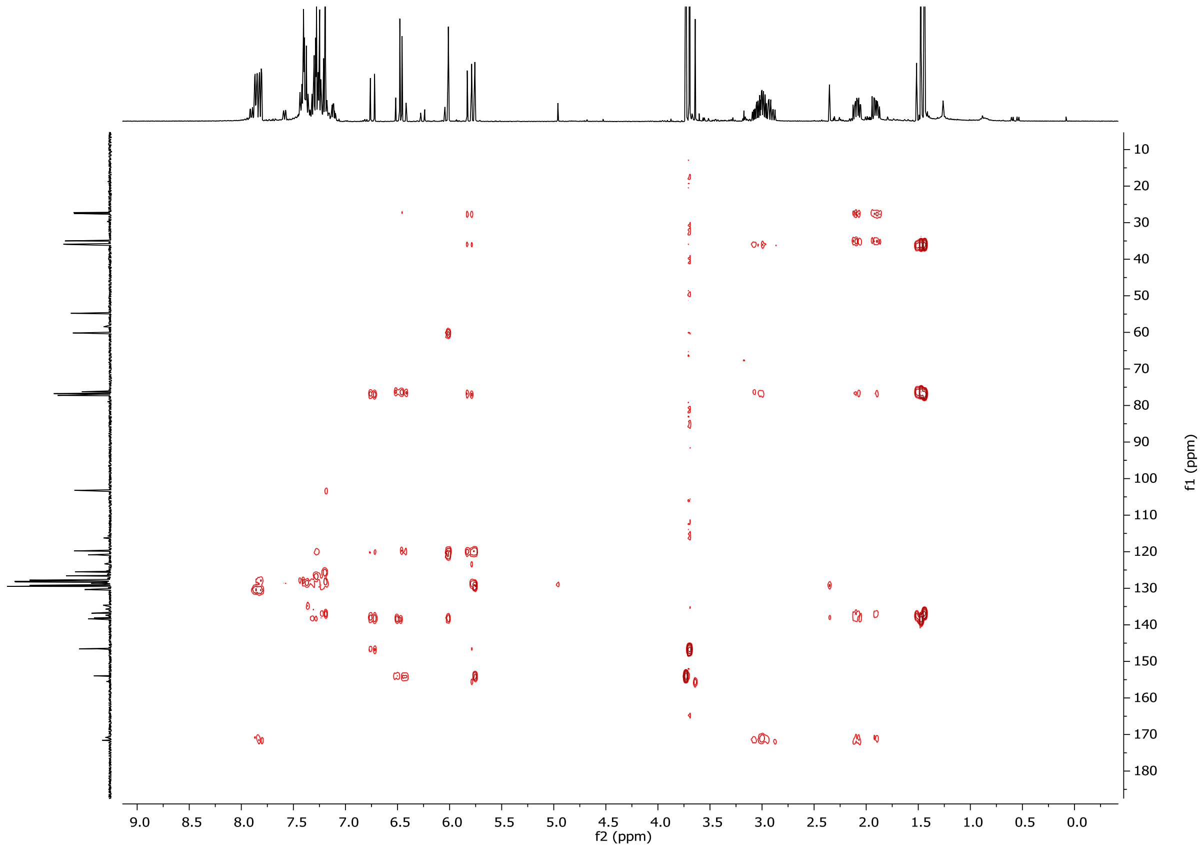
f2 (ppm)
f1 (ppm)

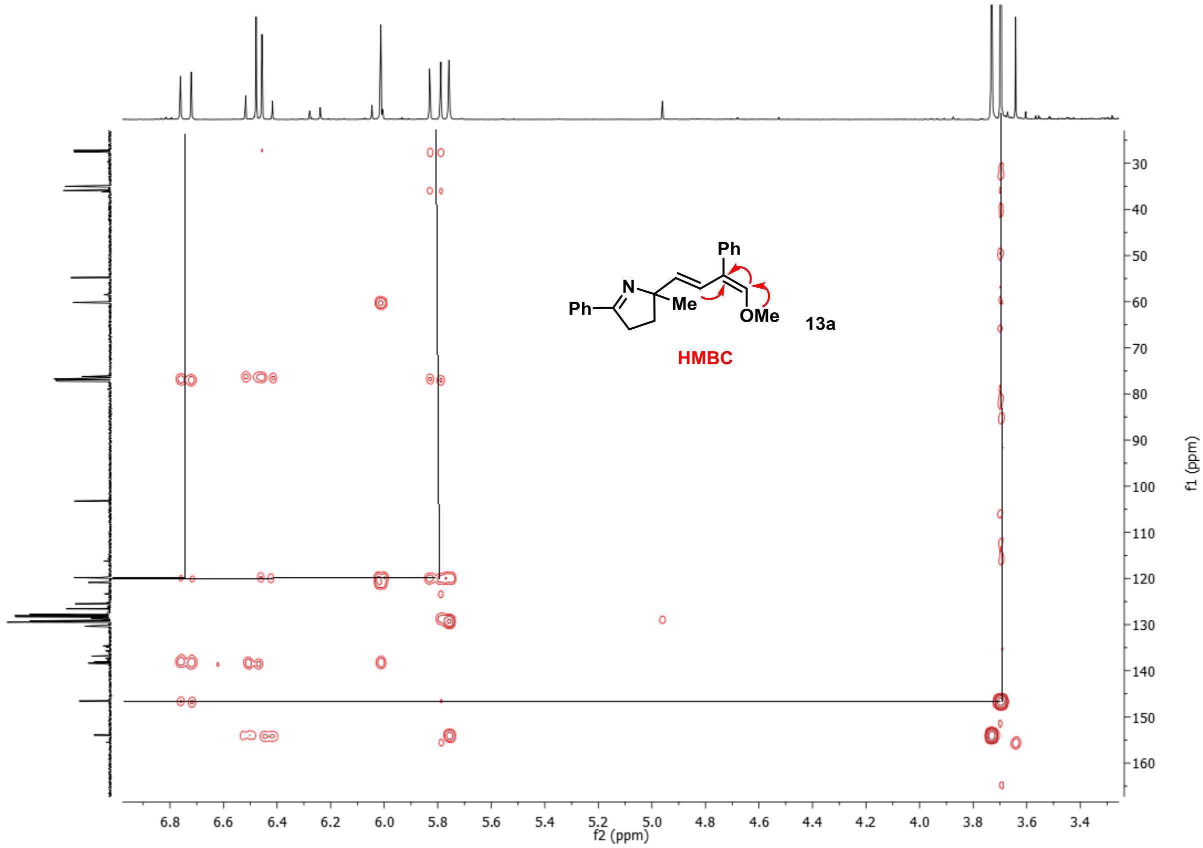
Ph
N
Me
Ph
OMe
13a
HMBC
f2 (ppm)
f1 (ppm)

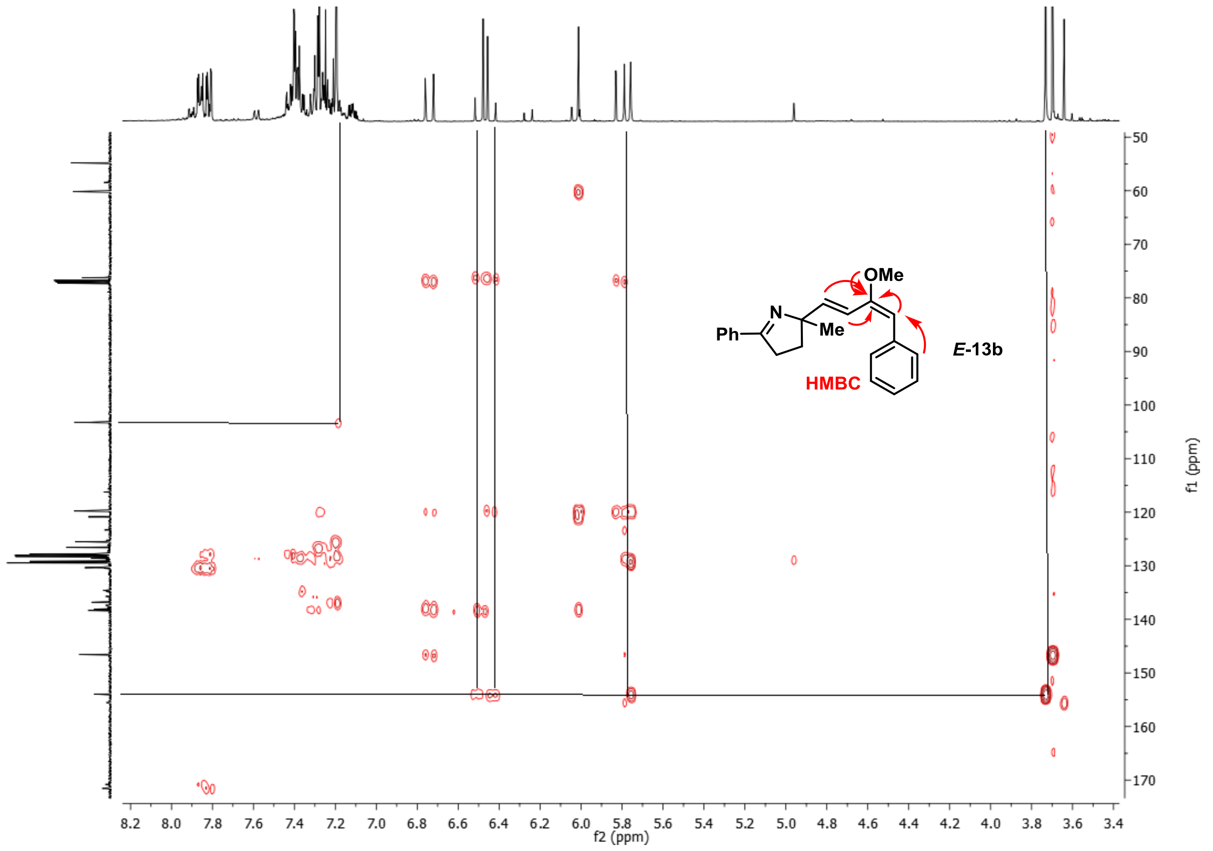

Ph
N
Me
OMe
HMBC
E-13b
f1 (ppm)
f2 (ppm)


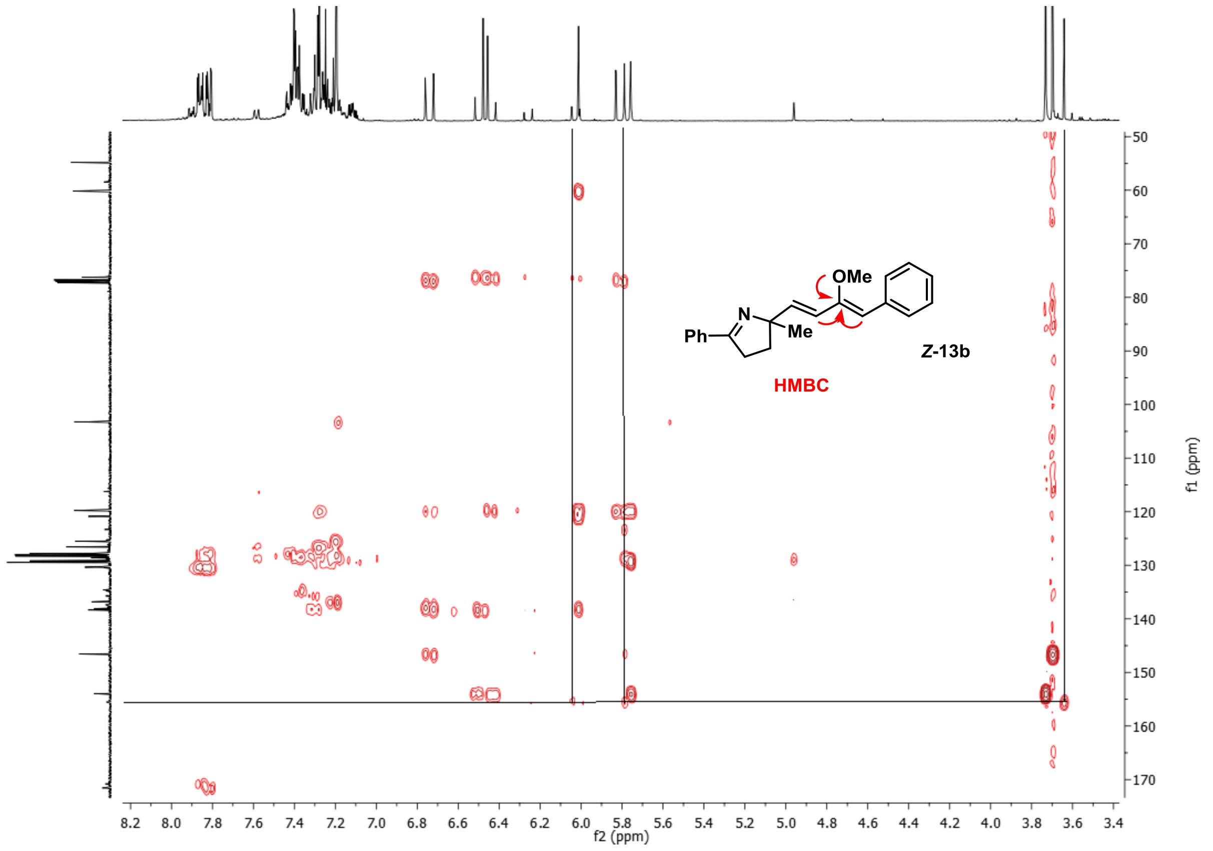

Ph
N
Me
OMe
HMBC
Z-13b
f1 (ppm)
f2 (ppm)

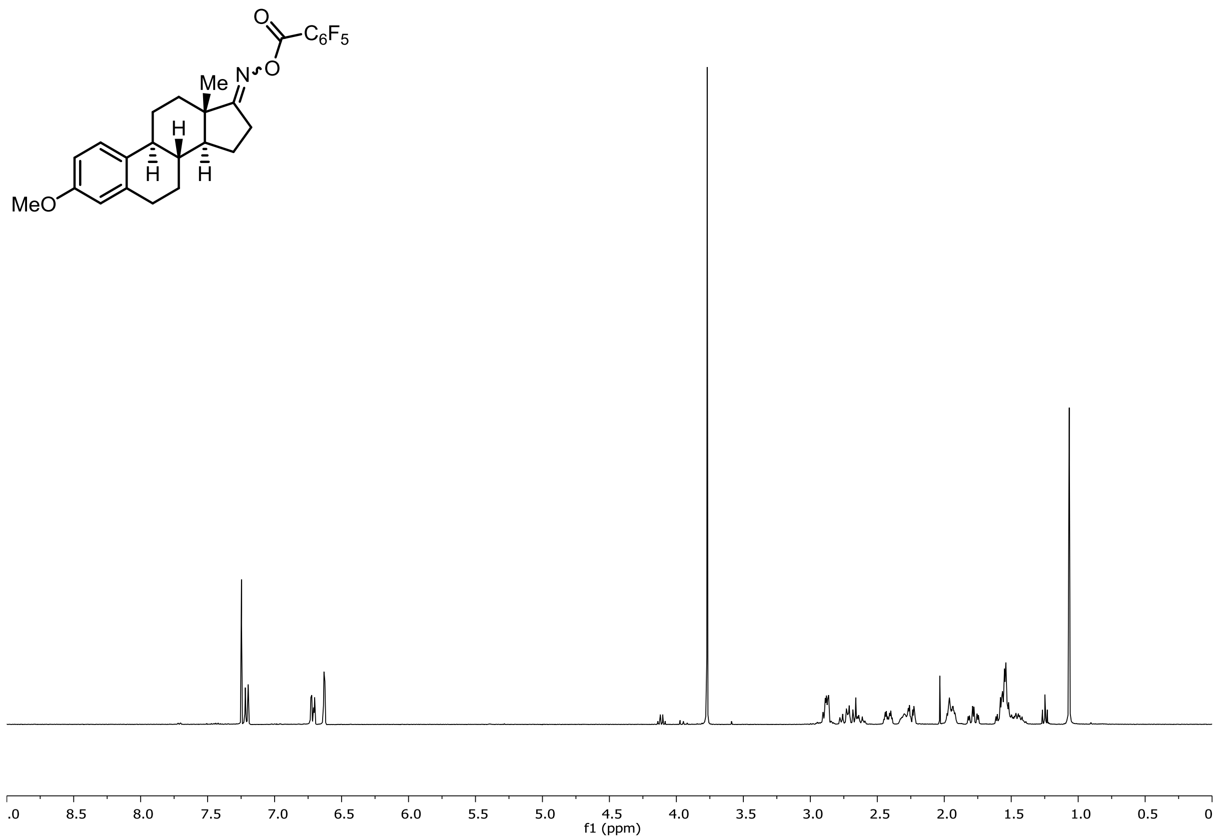
O
C6F5
N
O
Me
H
H
H
MeO
.0 8.5 8.0 7.5 7.0 6.5 6.0 5.5 5.0 4.5 4.0 3.5 3.0 2.5 2.0 1.5 1.0 0.5 0
f1 (ppm)

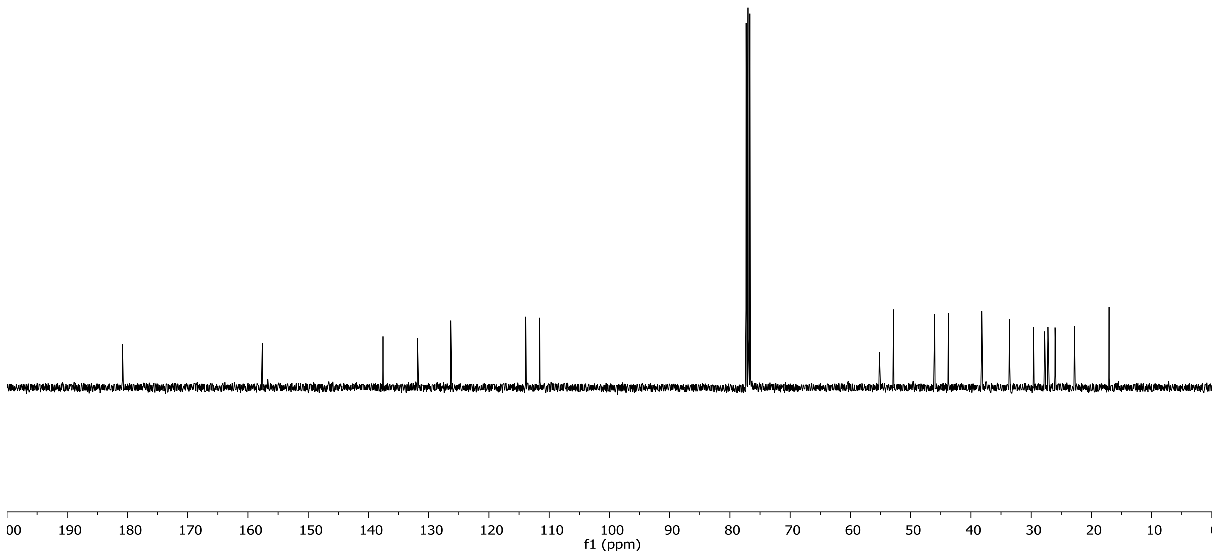
00 190 180 170 160 150 140 130 120 110 100 90 80 70 60 50 40 30 20 10
f1 (ppm)

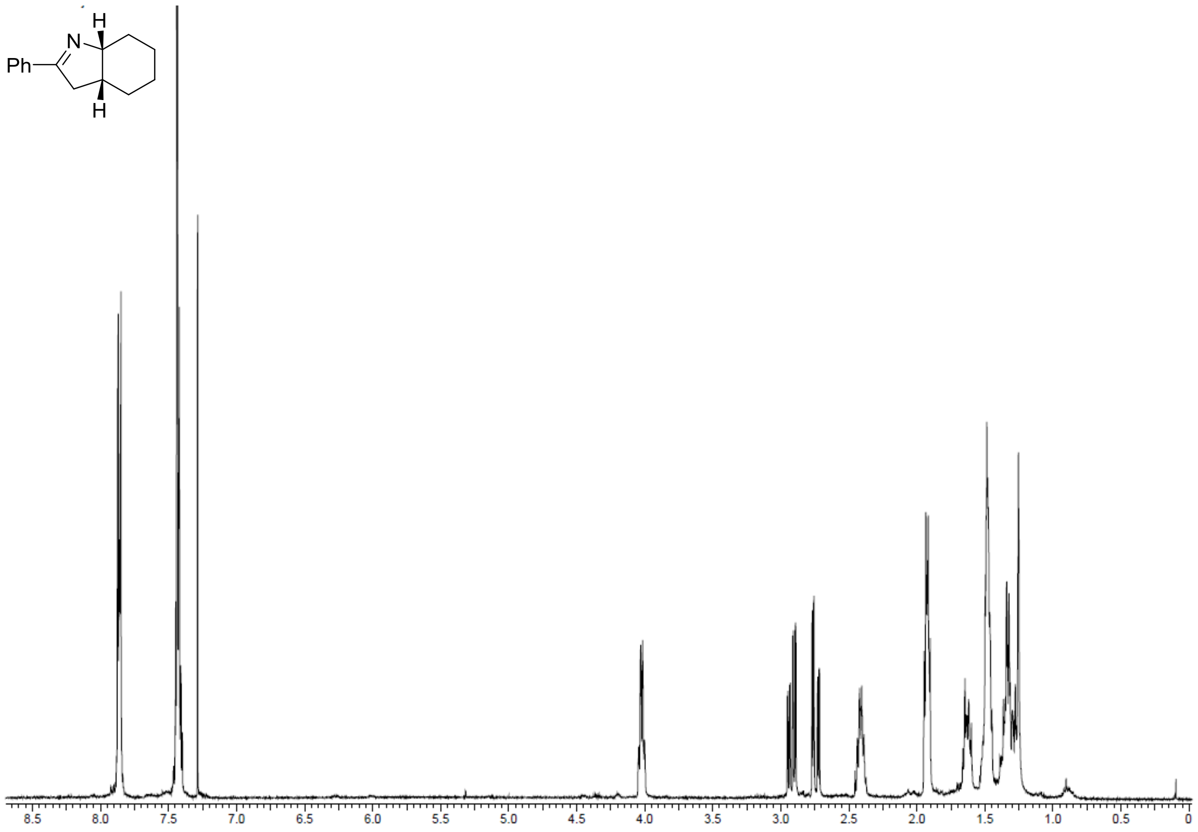
H
N
Ph
H

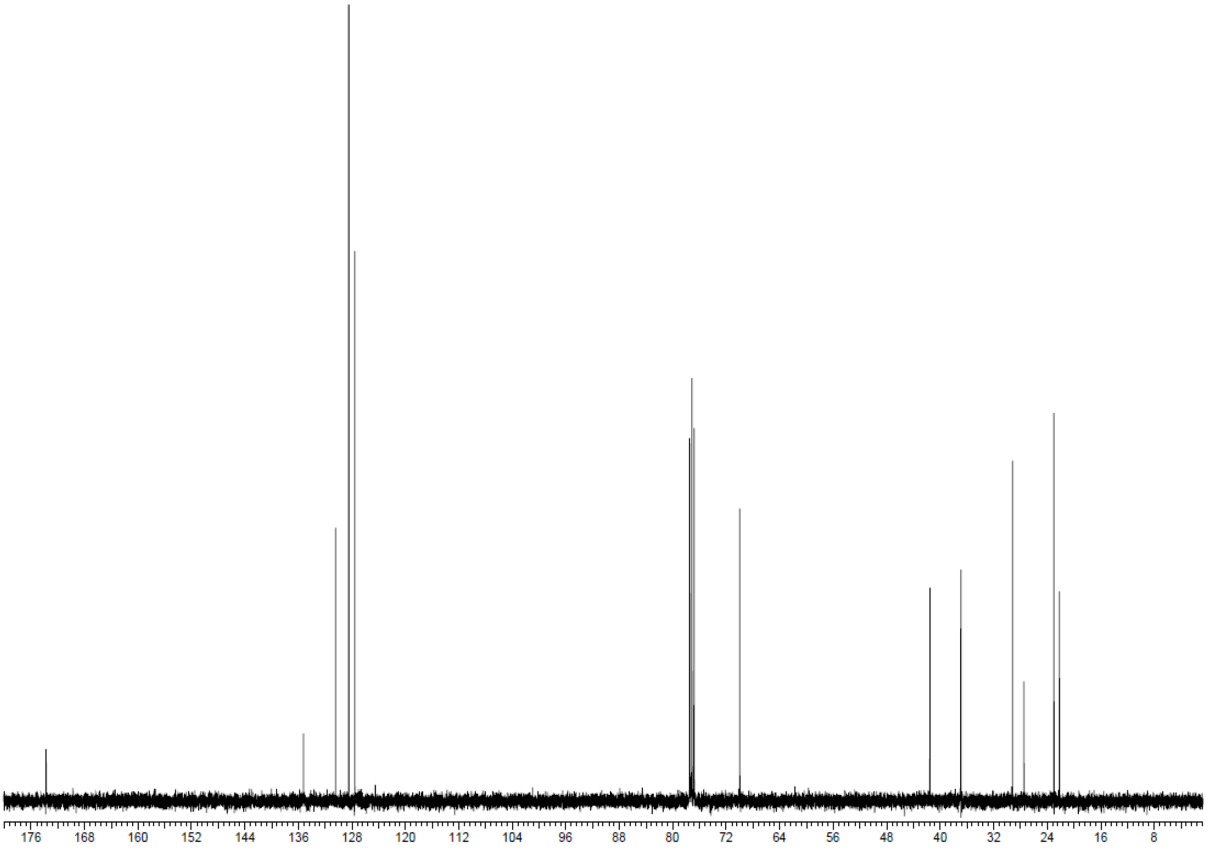

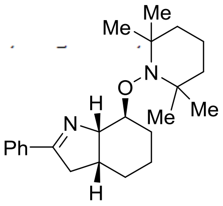
Me
Me
N
O
H
Me
Me
N
Ph
H

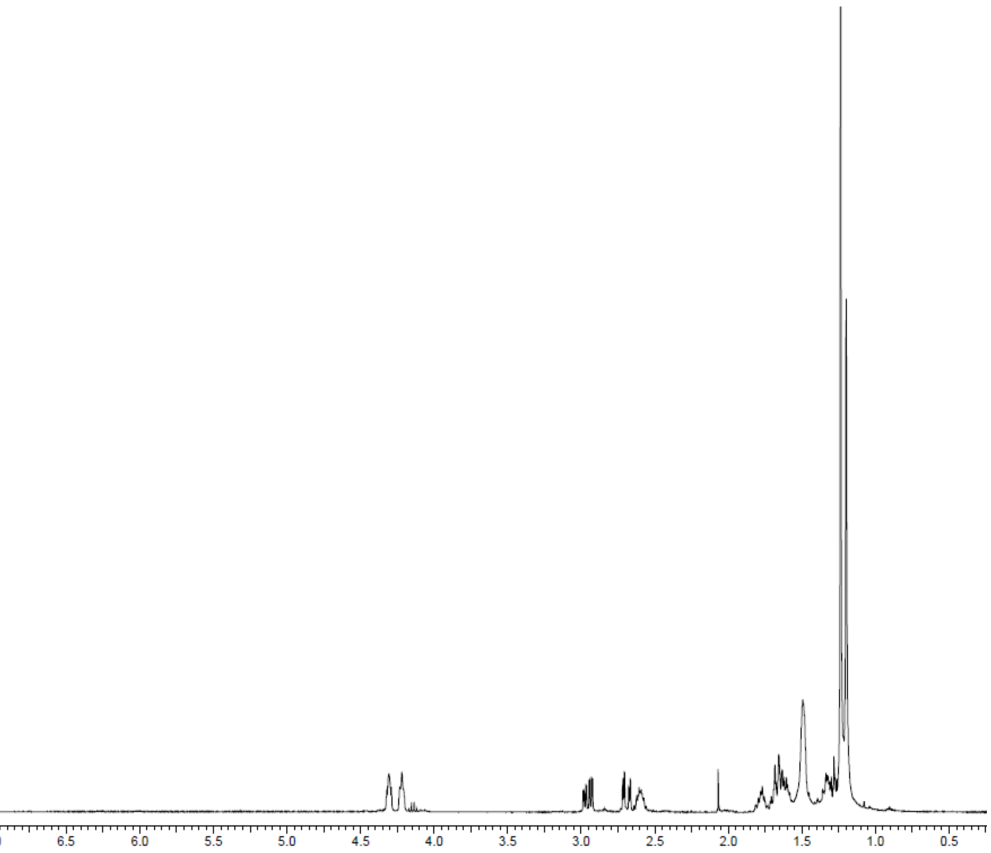
8.0 7.5 7.0 6.5 6.0 5.5 5.0 4.5 4.0 3.5 3.0 2.5 2.0 1.5 1.0 0.5

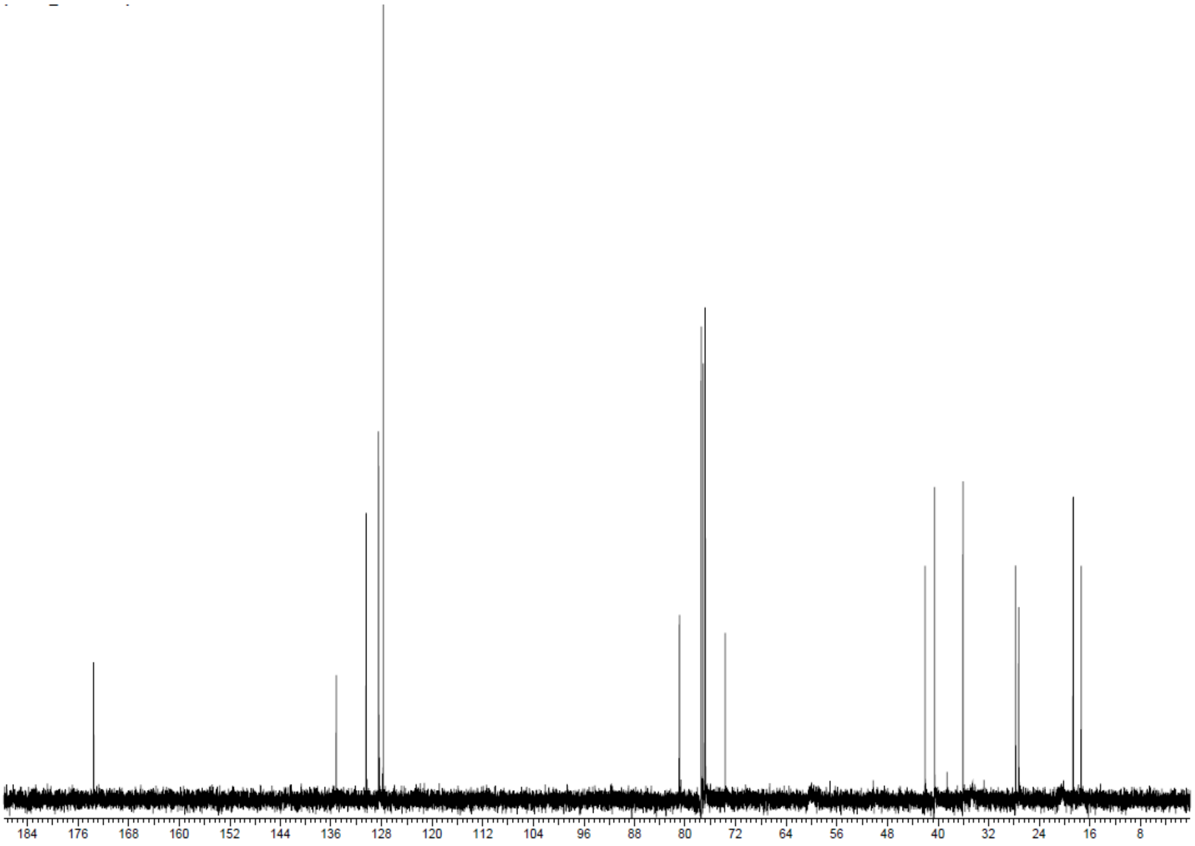
184 176 168 160 152 144 136 128 120 112 104 96 88 80 72 64 56 48 40 32 24 16 8

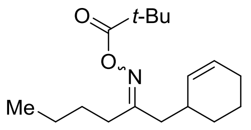
O
t-Bu
O
N
Me

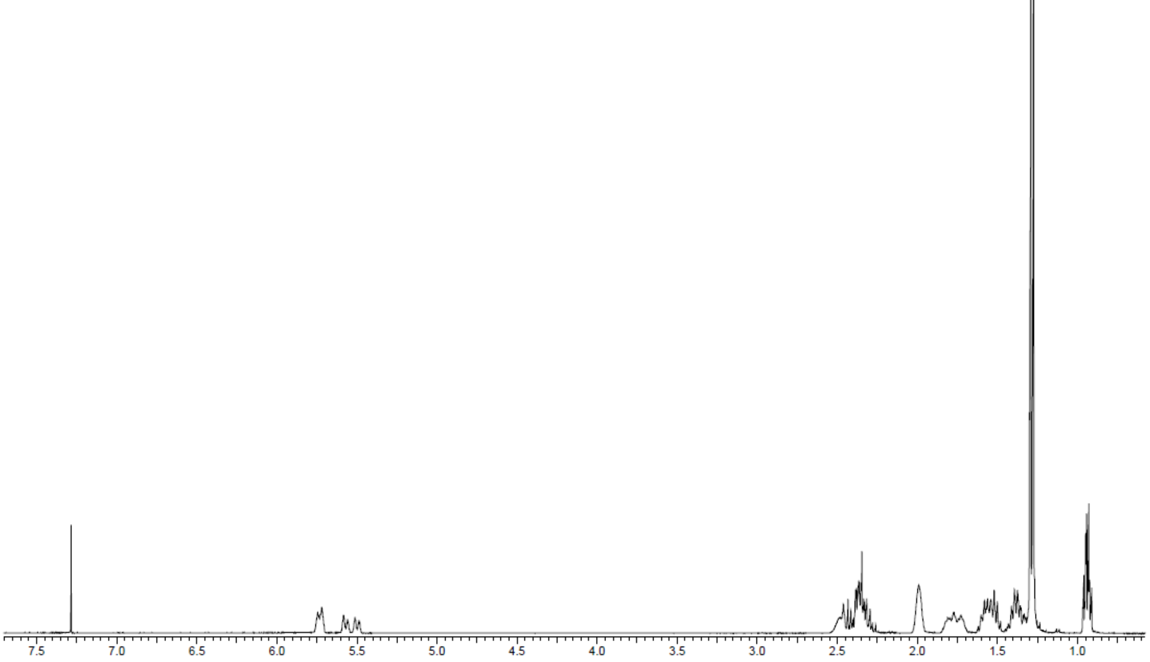

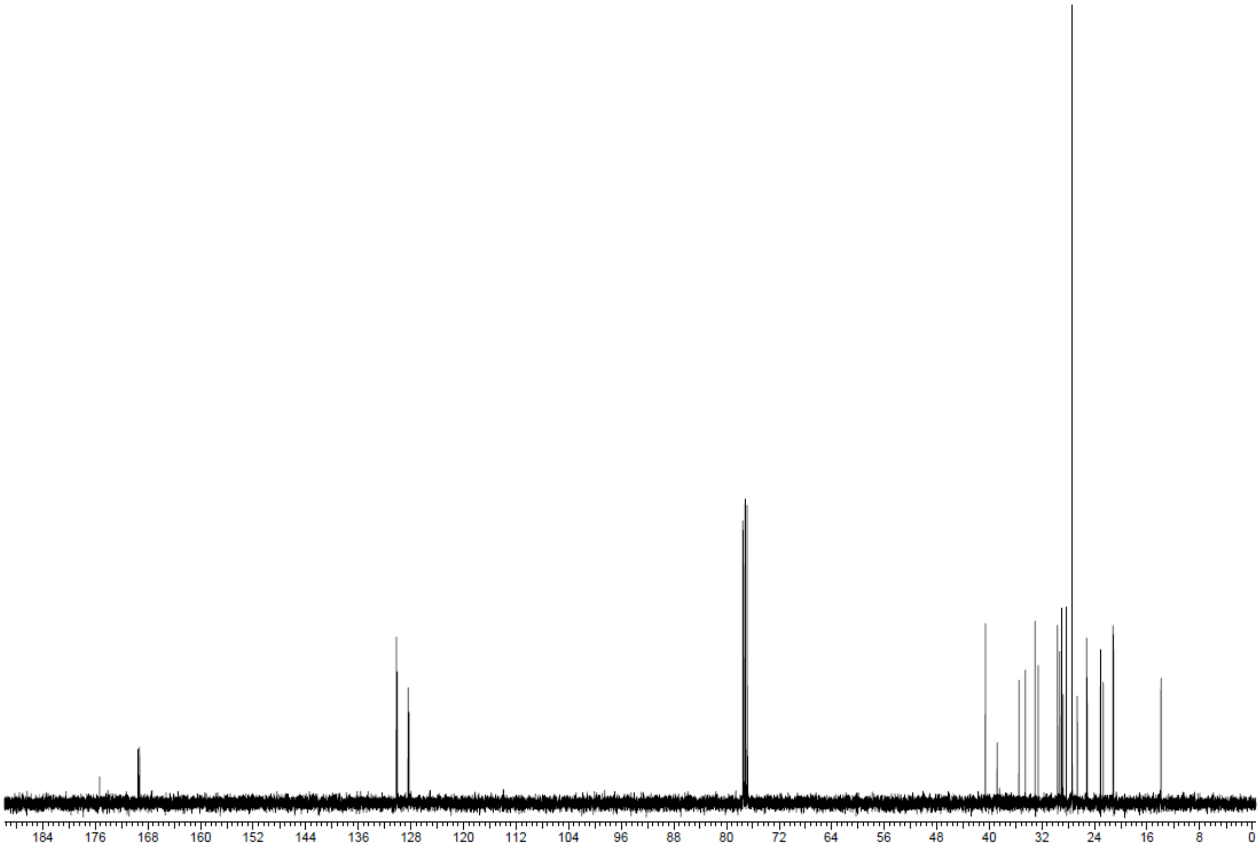

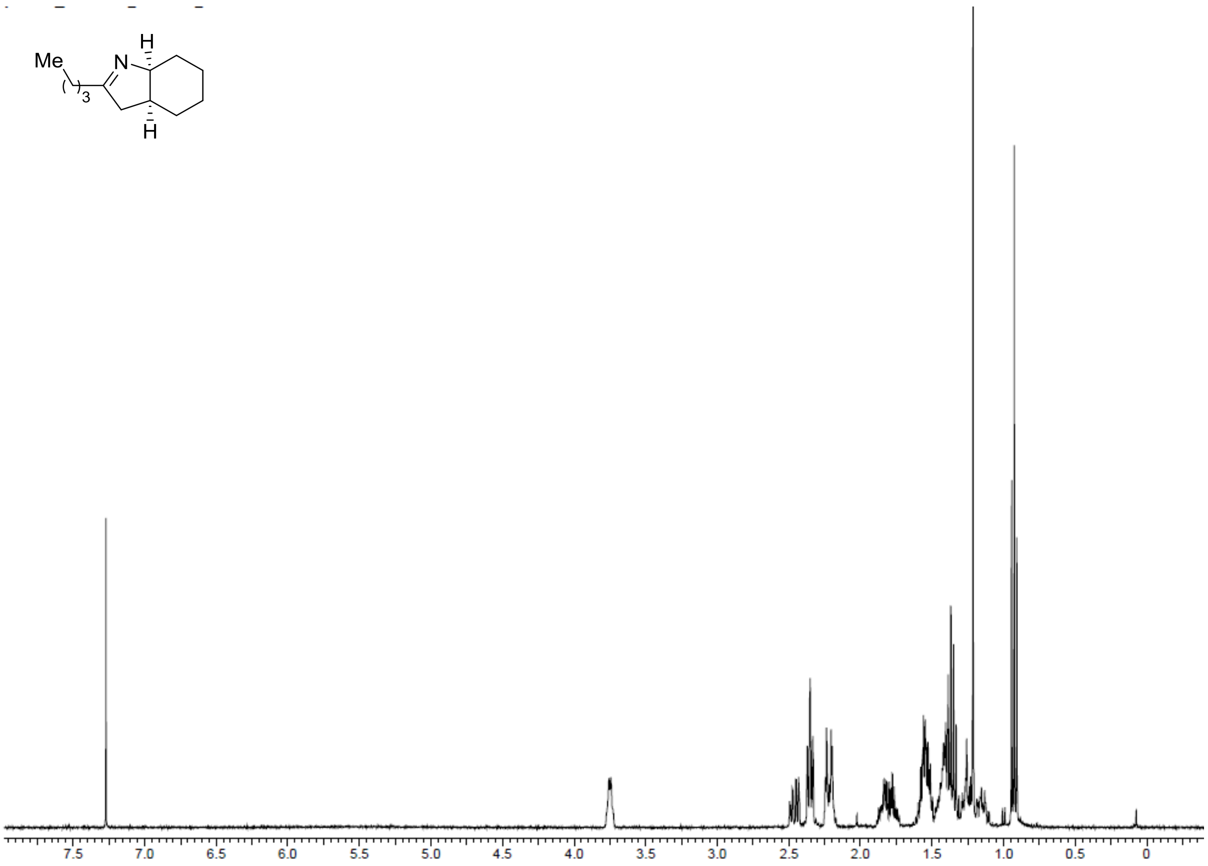
Me
N
H
H
3
7.5 7.0 6.5 6.0 5.5 5.0 4.5 4.0 3.5 3.0 2.5 2.0 1.5 1.0 0.5 0

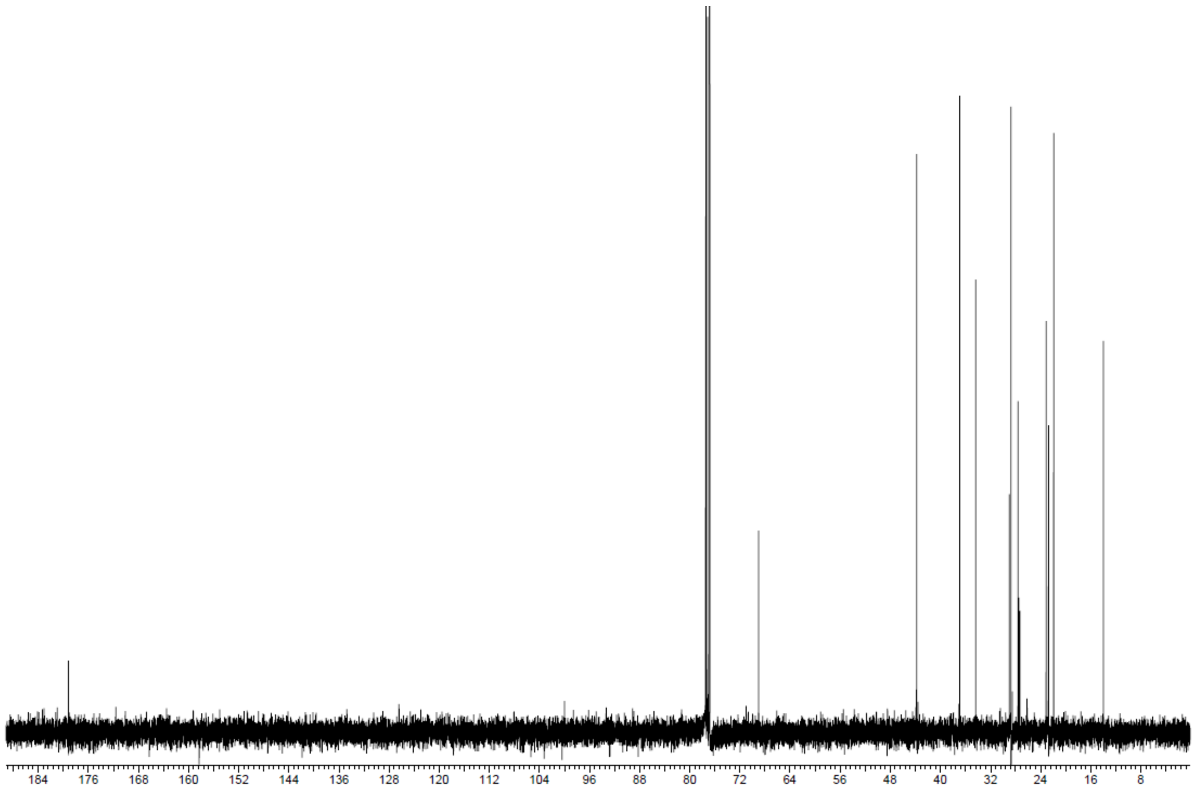
184 176 168 160 152 144 136 128 120 112 104 96 88 80 72 64 56 48 40 32 24 16 8

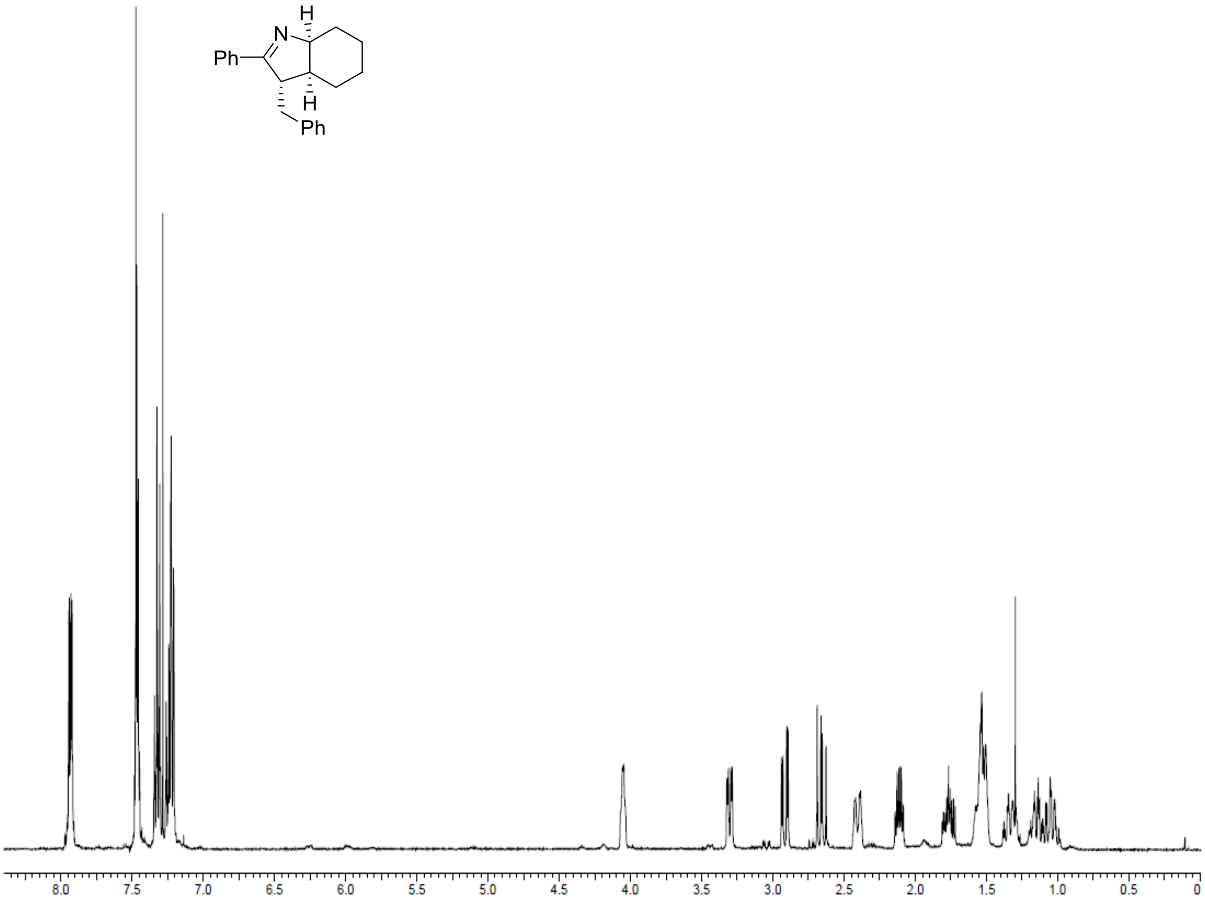

Ph
N
H
H
Ph
8.0 7.5 7.0 6.5 6.0 5.5 5.0 4.5 4.0 3.5 3.0 2.5 2.0 1.5 1.0 0.5 0


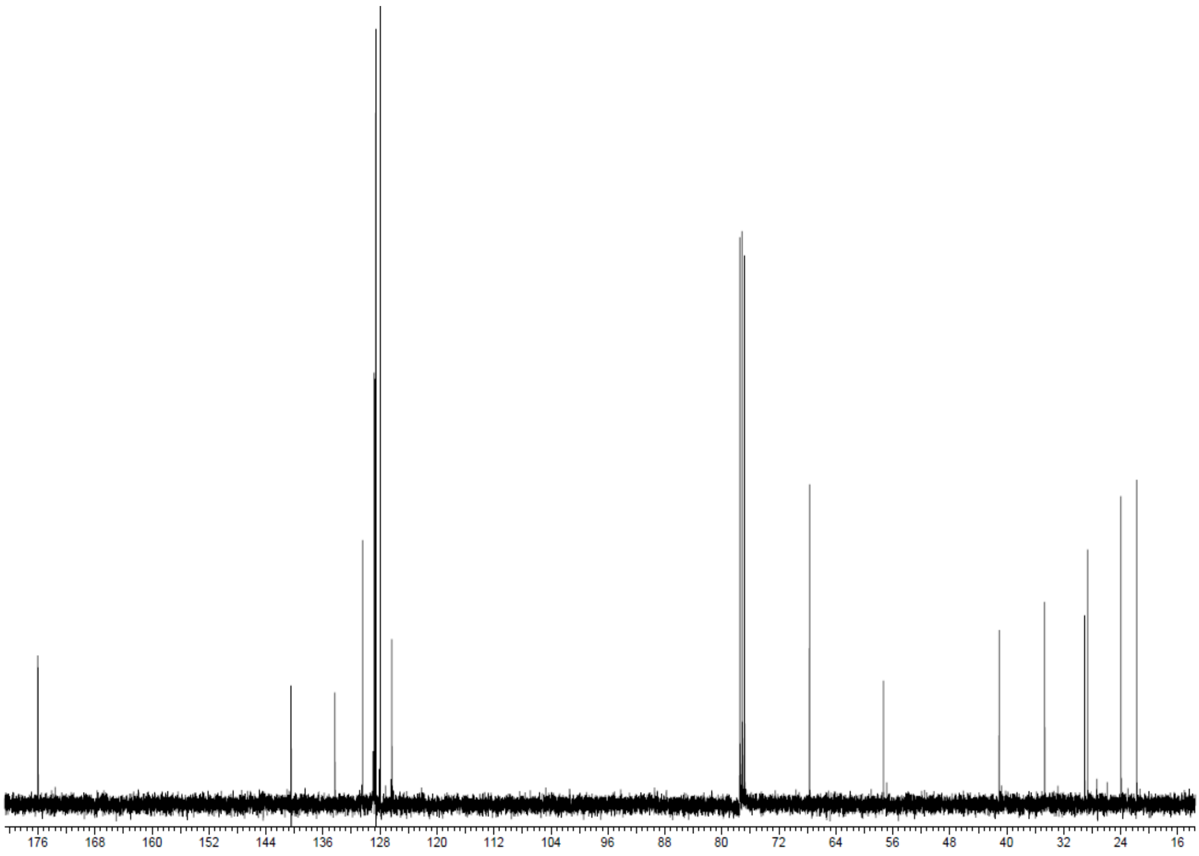

176 168 160 152 144 136 128 120 112 104 96 88 80 72 64 56 48 40 32 24 16

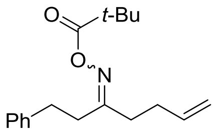
O
t-Bu
O
N
Ph

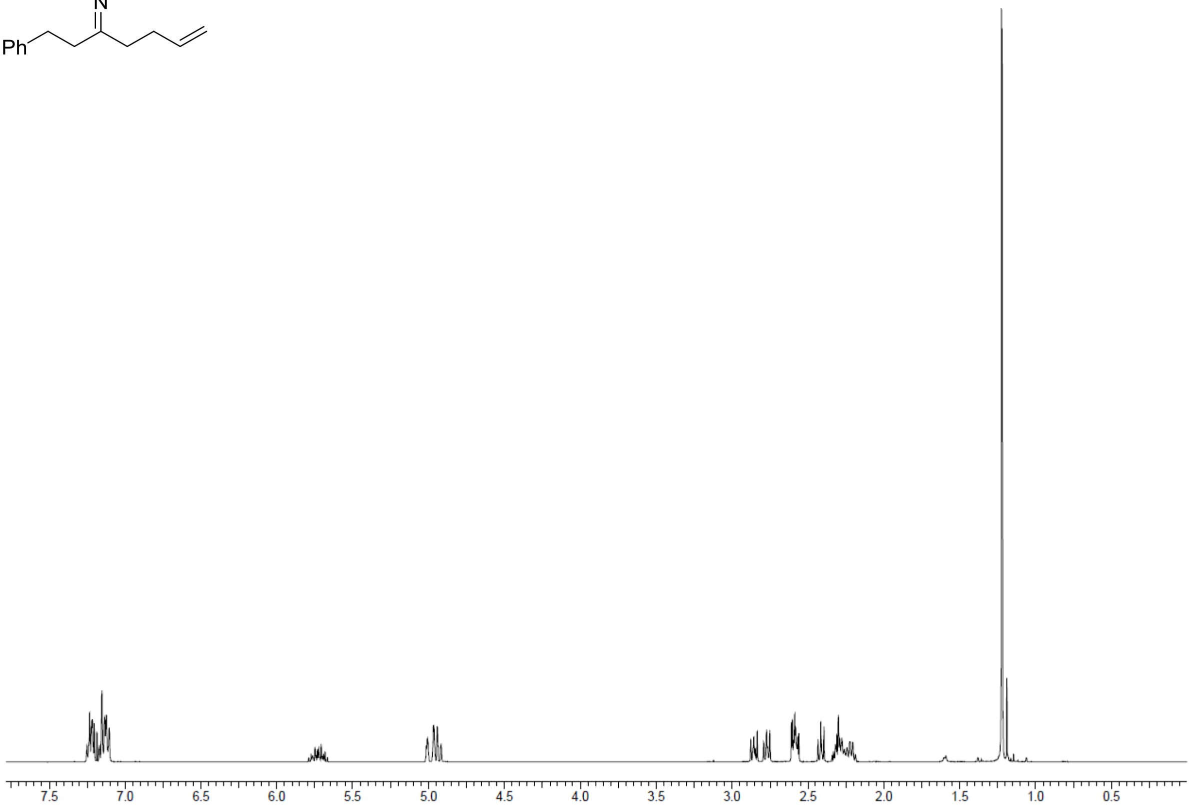

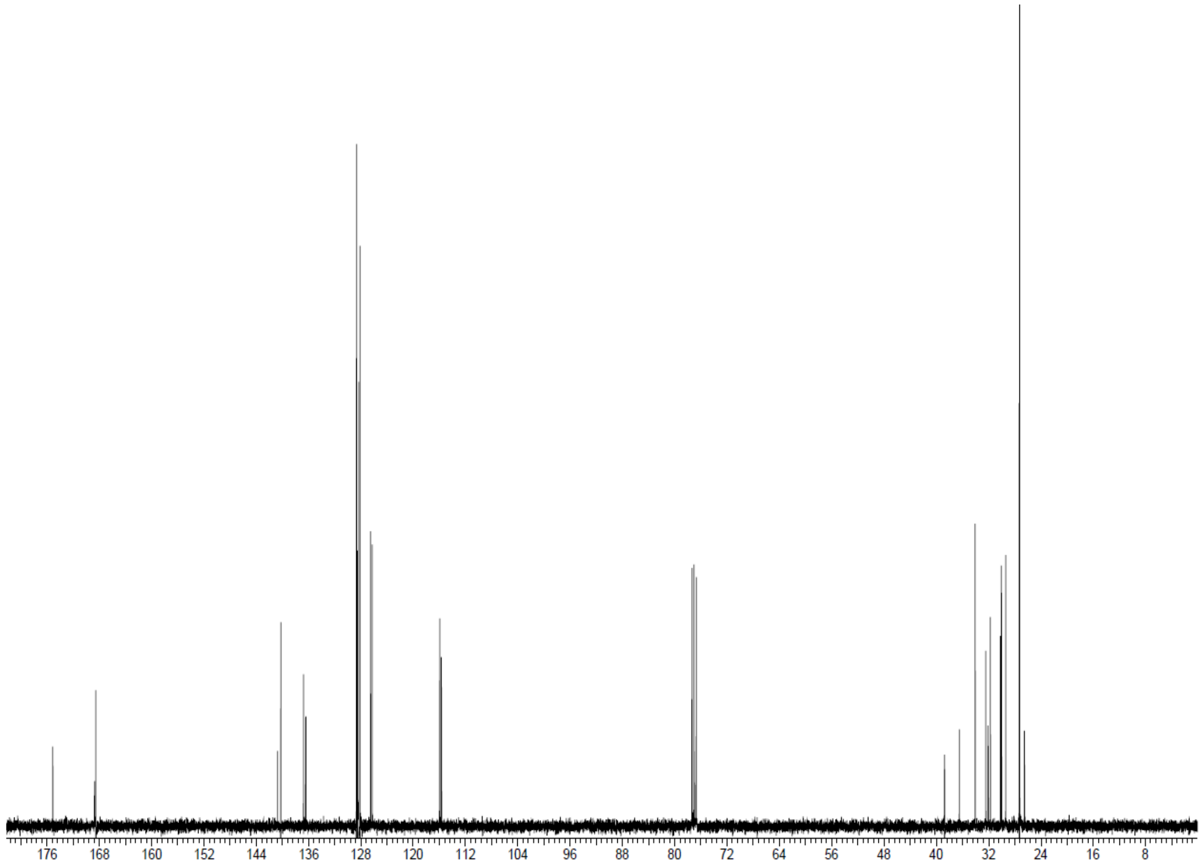

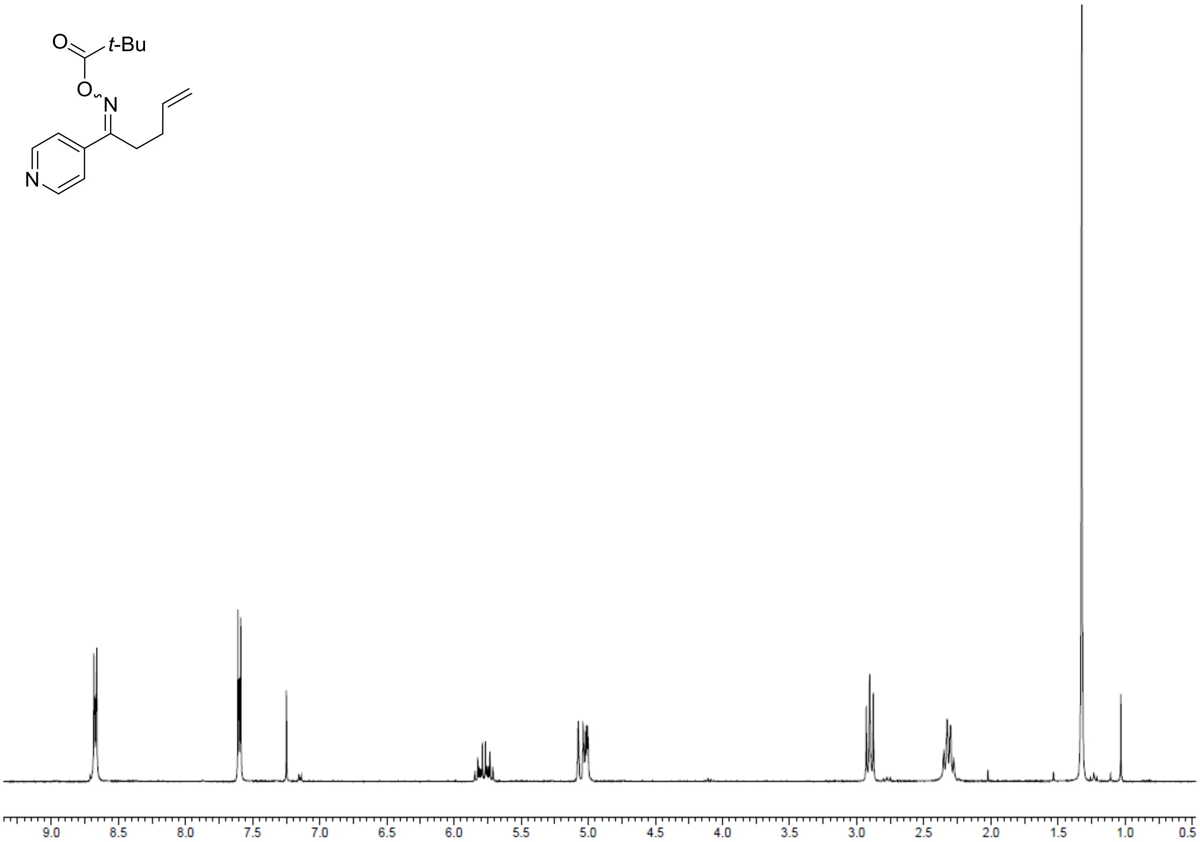
O
t-Bu
O
N
N
9.0 8.5 8.0 7.5 7.0 6.5 6.0 5.5 5.0 4.5 4.0 3.5 3.0 2.5 2.0 1.5 1.0 0.5

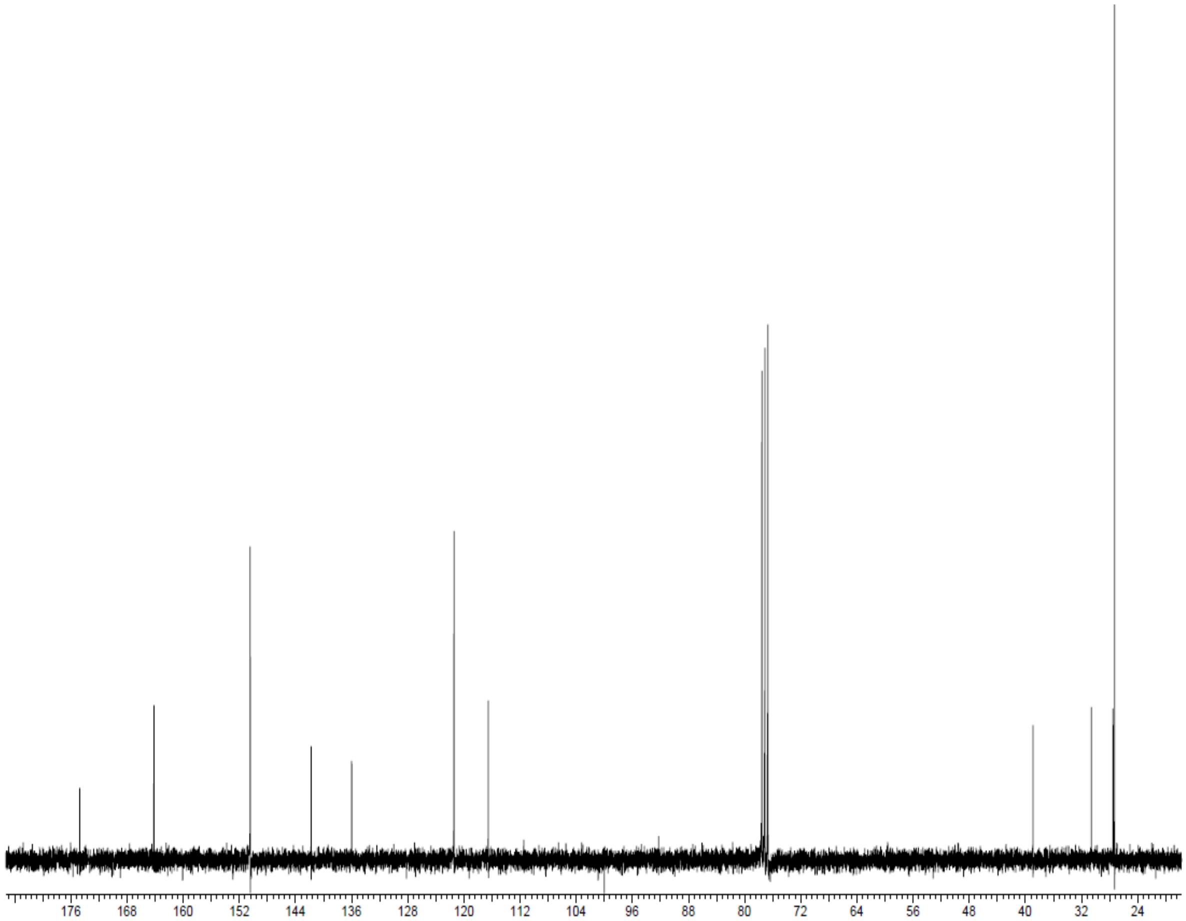
176 168 160 152 144 136 128 120 112 104 96 88 80 72 64 56 48 40 32 24

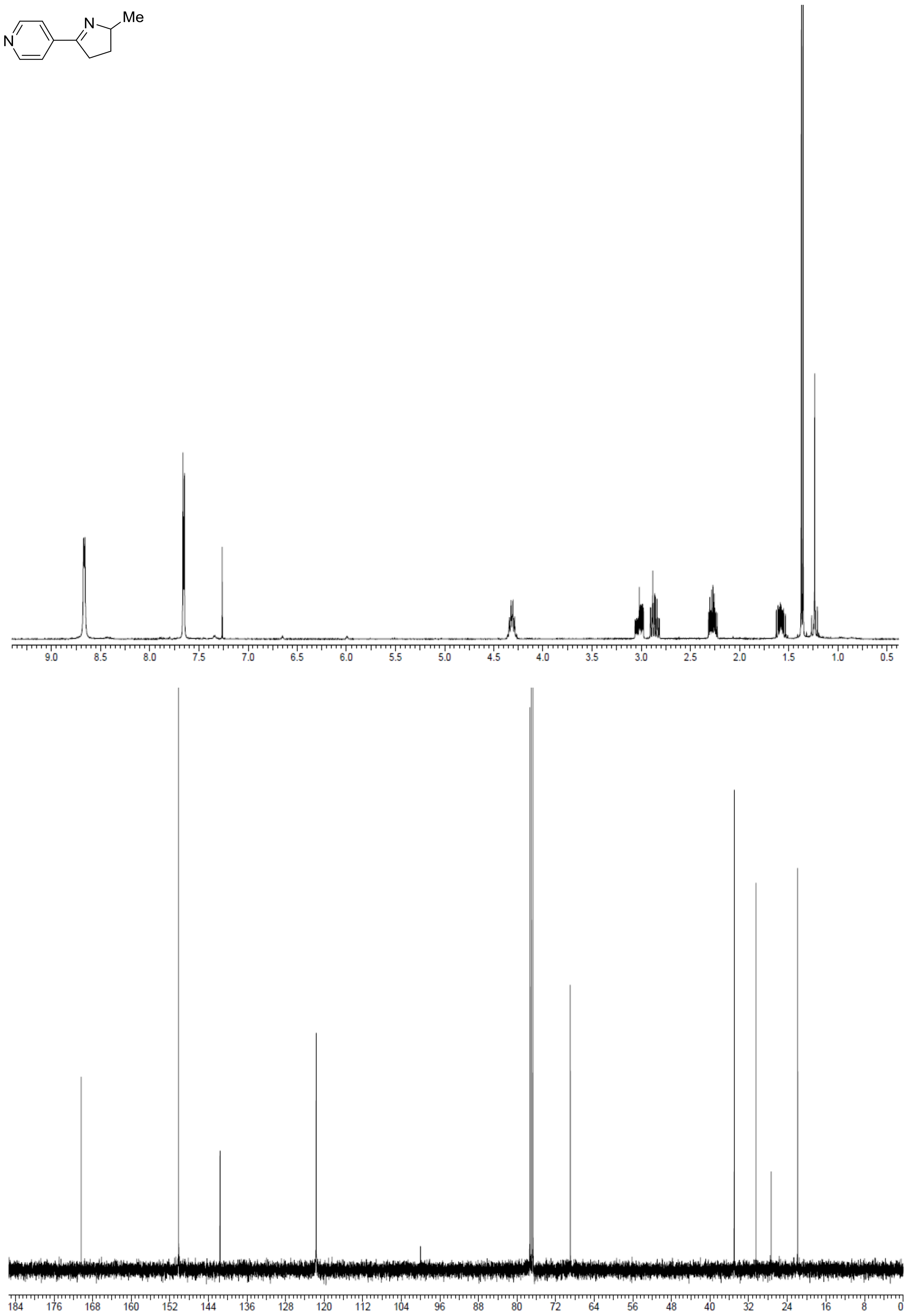
N
N
Me

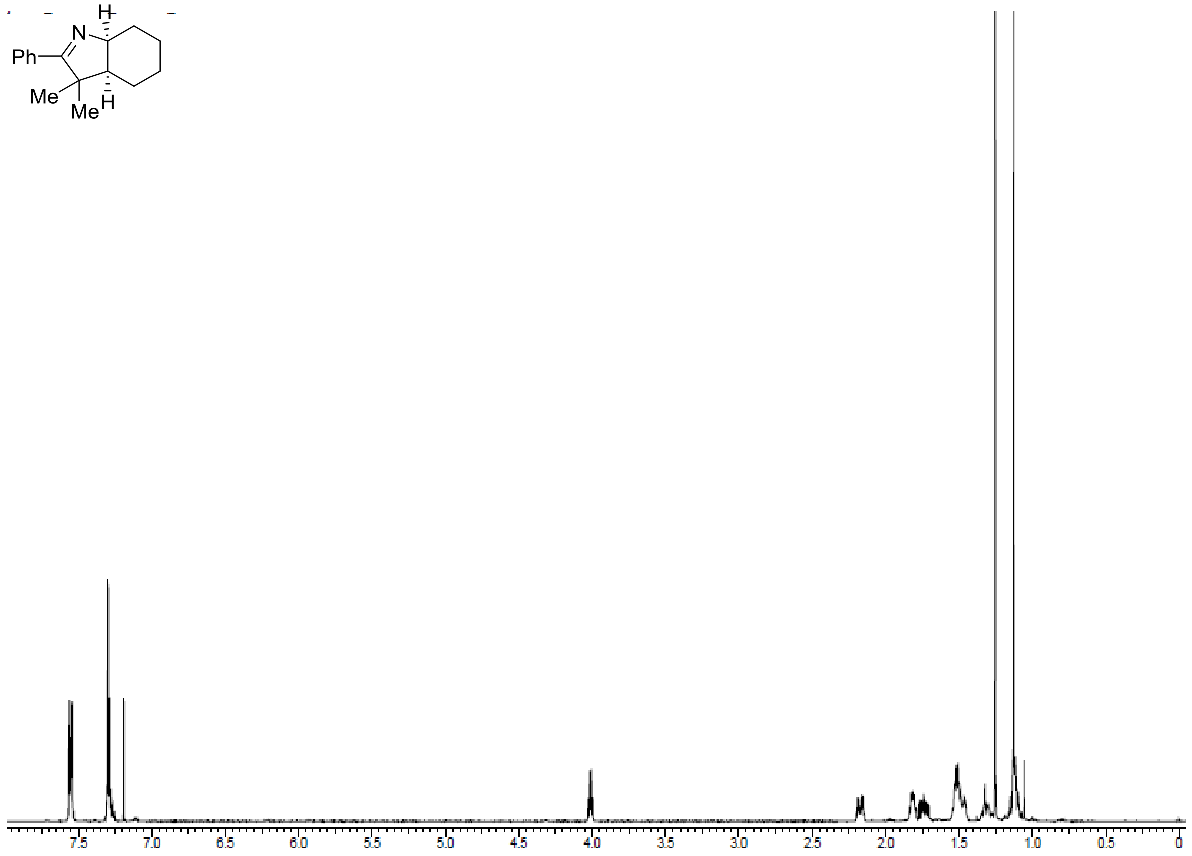
H
N
Ph
Me
Me
H
7.5 7.0 6.5 6.0 5.5 5.0 4.5 4.0 3.5 3.0 2.5 2.0 1.5 1.0 0.5 0

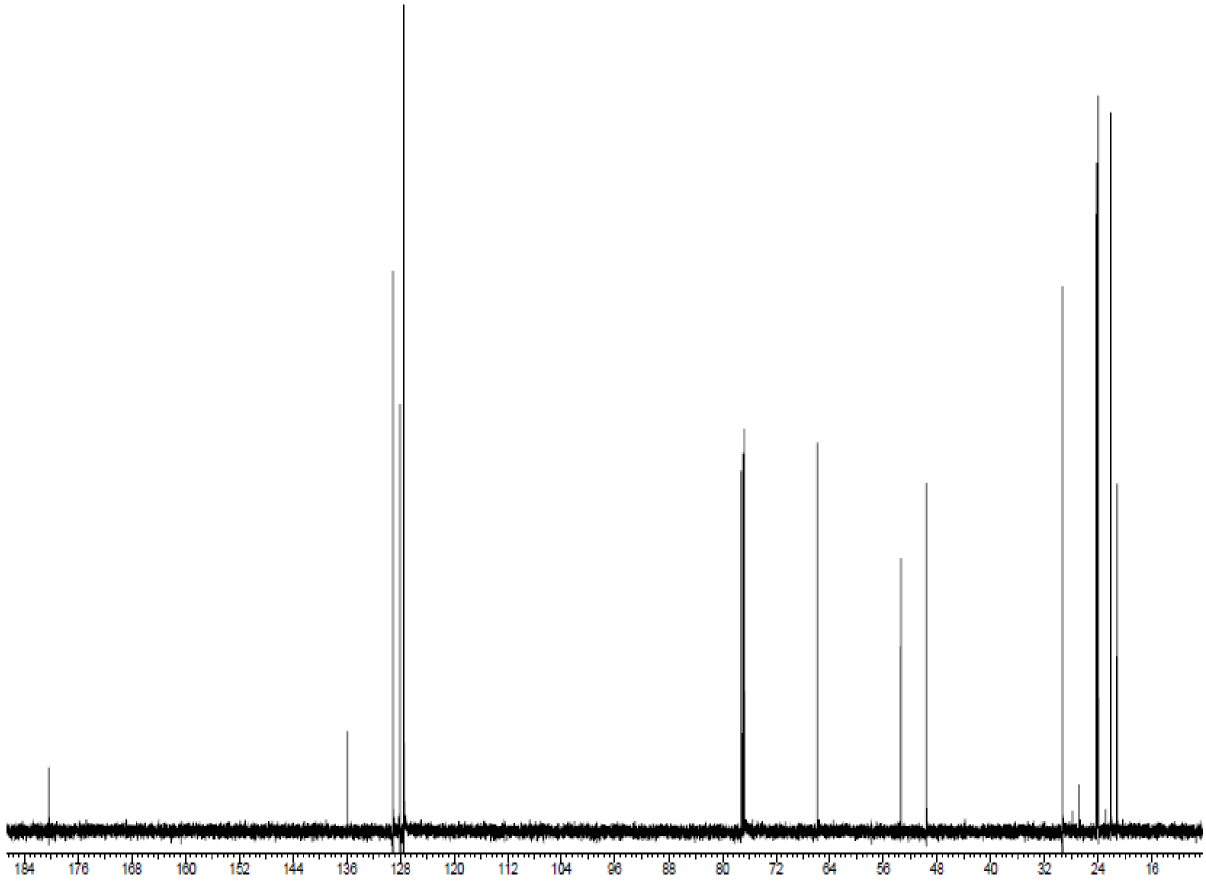
184 176 168 160 152 144 136 128 120 112 104 96 88 80 72 64 56 48 40 32 24 16

## References

(1) Faulkner, A.; Bower, J. F. *Angew. Chem. Int. Ed.* **2012**, *51*, 1675.
(2) Faulkner, A.; Race, N. J.; Bower, J. F. *Chem. Sci.* **2014**, *5*, 2416.
(3) Rukachaisirikul, V.; Koert, U.; Reinhard, W. H. *Tetrahedron* **1992**, *48*, 4533.
(4) Kitamura, M.; Yoshida, M.; Kikuchi, T.; Narasaka, K *Synthesis* **2003**, 2415.
(5) Uchiyama, K.; Hayashi, Y.; Narasaka, K. *Tetrahedron*, **1999**, *55*, 8915.
(6) Zhu, J-L.; Su, Y-L.; Chan, Y-H.; Chen, I-C.; Liao, C-C. *Heterocycles*, **2009**, *78*, 369.
